# Matching circadian rhythms to light–dark cycles increases lettuce yield by 29% in vertical farms without additional energy input

**DOI:** 10.1093/jxb/erag222

**Published:** 2026-05-12

**Authors:** Cèlia Anton-Sales, Louise Benckhuysen, Bünyamin Peker, Marieke Jeuken, Guusje Bonnema

**Affiliations:** Plant Breeding, Wageningen University and Research, Wageningen, The Netherlands; Plant Breeding, Wageningen University and Research, Wageningen, The Netherlands; Avery Family Farms, Okanagan Falls, British Columbia, Canada; Plant Breeding, Wageningen University and Research, Wageningen, The Netherlands; Plant Breeding, Wageningen University and Research, Wageningen, The Netherlands; Plant Breeding, Wageningen University and Research, Wageningen, The Netherlands; University of Glasgow, UK

**Keywords:** Circadian clock, circadian resonance, controlled-environment agriculture, *Lactuca sativa*, sustainable horticulture, vertical farming, yield optimization

## Abstract

Most cultivated lettuce varieties have 27 h circadian clocks resulting from domestication-driven selection for delayed bolting yet are grown under standard 24 h light–dark cycles. This creates a fundamental mismatch between plant biology and cultivation practices. According to circadian resonance theory, this misalignment imposes chronic re-entrainment costs that reduce fitness. Here we demonstrate that eliminating this mismatch through circadian resonance, namely aligning environmental cycles with internal rhythms, increases lettuce biomass by 11–29% without additional lighting energy in commercial vertical farming conditions. We grew 20 lettuce accessions with characterized circadian periods (24–28 h) under six light–dark treatments combining two cycle lengths (24 h and 27 h) with three photoperiod regimes. Long-clock varieties (∼27 h) consistently accumulated more biomass under 27 h versus 24 h cycles when the daily light integral remained constant (14.4 mol m^−2^ d^−1^). Enhanced yields resulted from extended dark periods (11 h versus 8 h) rather than increased light input, representing a lighting-efficient cultivation strategy. High-energy treatments (18 mol m^−2^ d^−1^) achieved maximum biomass but triggered morphological aberrations and increased tipburn, while resonant conditions maintained quality standards. Resonant conditions also restored natural developmental timing, with long-clock varieties showing accelerated bolting that reversed the delayed flowering phenotype observed under 24 h cycles. Importantly, bolting still falls outside the cultivation window. Statistical modelling confirmed significant interactions between the circadian clock and the diel length, establishing circadian resonance as the primary driver of yield improvement. These findings reveal circadian resonance as a transformative strategy for yield enhancement in controlled-environment agriculture, such as vertical farms, achieving significant biomass gains through biological rhythm optimization.

## Teaser

Most cultivated lettuce varieties have a 27 h internal clock but are grown under 24 h light cycles; we confirm circadian resonance effects in a crop species and demonstrate yield increases of 11–29%.

## Introduction

Circadian clocks evolved to synchronize biological processes with Earth’s 24 h rotation, enabling organisms to anticipate and efficiently respond to predictable daily environmental changes (Harmer *et al.*, 2001). These internal timekeeping mechanisms regulate nearly all biological processes through transcriptional–translational feedback loops that generate autonomous ∼24 h rhythms (McClung, 2006, 2019). These periodic and autonomous oscillations are referred to as free running, since they are sustained without any environmental input (i.e. they keep occurring every ∼24 h in complete darkness).

The circadian resonance hypothesis, proposed by Pittendrigh and Minis (1972), states that fitness maximizes when endogenous periods (tau, τ) synchronize with environmental cycles. Thus, when internal periods (≠24 h) deviate from external cycles (24 h), organisms face chronic re-entrainment costs that reduce fitness. Supporting evidence spans multiple kingdoms: *Drosophila* and blowflies show extended life spans under matched cycles (Pittendrigh and Minis, 1972; von Saint Paul and Aschoff, 1978), primates exhibit improved survival under 24 h days (Hozer *et al.*, 2020; Hozer and Pifferi, 2020), cyanobacteria demonstrate enhanced growth under resonant conditions (Ouyang *et al.*, 1998), and Arabidopsis plants grown under non-matching photoperiods show reduced chlorophyll, growth, and survival (Dodd *et al.*, 2005).

Our previous work has shown that most cultivated lettuce (*Lactuca sativa*) varieties possess decelerated circadian clocks with periods of ∼27 h (Anton-Sales *et al.*, 2025). This clock alteration arose through domestication-driven selection for delayed bolting via a truncating mutation in the *PHYTOCHROME C* (*PHYC*) locus, which simultaneously slowed the circadian oscillator and postponed bolting and flowering (Anton-Sales *et al.*, 2025). While this trait extends the vegetative growth phase under field conditions, it creates a fundamental mismatch: plants with 27 h internal clocks are cultivated under standard 24 h light–dark cycles in fields, greenhouses, and controlled-environment systems.

In plants, the circadian clock serves as a master regulator of agronomically important traits including flowering time, photosynthesis, and carbohydrate metabolism (Pittendrigh and Minis, 1964; Graf *et al.*, 2010; Haydon *et al.*, 2013; Yang *et al.*, 2024). One key mechanism is the circadian control of starch degradation, which sustains growth during darkness by ensuring energy reserves are neither depleted prematurely nor left unused (Graf *et al.*, 2010; Pilkington *et al.*, 2015). This precise coordination represents a physiological mechanism potentially underlying growth benefits observed under circadian resonance (Graf *et al.*, 2010). Thus, the theoretical basis of circadian resonance depends on energetic costs: organisms with free-running periods deviating from 24 h require daily re-entrainment to external time cues, incurring chronic physiological and metabolic expenses (Jabbur *et al.*, 2024).

Despite broad taxonomic support, circadian resonance remains largely unexplored in crop production. Most studies have focused on model organisms under laboratory conditions, with limited systematic investigation in crops under commercial growing environments. This gap persists due to challenges in measuring circadian periods in diverse germplasm and implementing non-24 h cycles at production scale.

Lettuce represents an ideal system to test circadian resonance effects in a crop species. Our previous characterization revealed that most cultivated lettuce accessions exhibit 27 h clocks, while a small segment of cultivated lettuce represented mostly by winter types and wild relatives retain 24 h rhythms (Anton-Sales *et al.*, 2025). This variation, combined with the suitability of lettuce for controlled-environment agriculture (CEA), creates unique opportunities to test whether matching cultivation cycles to internal clocks can improve productivity. Explicitly, vertical farming facilities enable precise manipulation of photoperiod and cycle length impossible in field or greenhouse settings, allowing rigorous testing of circadian resonance under commercial production conditions.

**Fig. 1.**
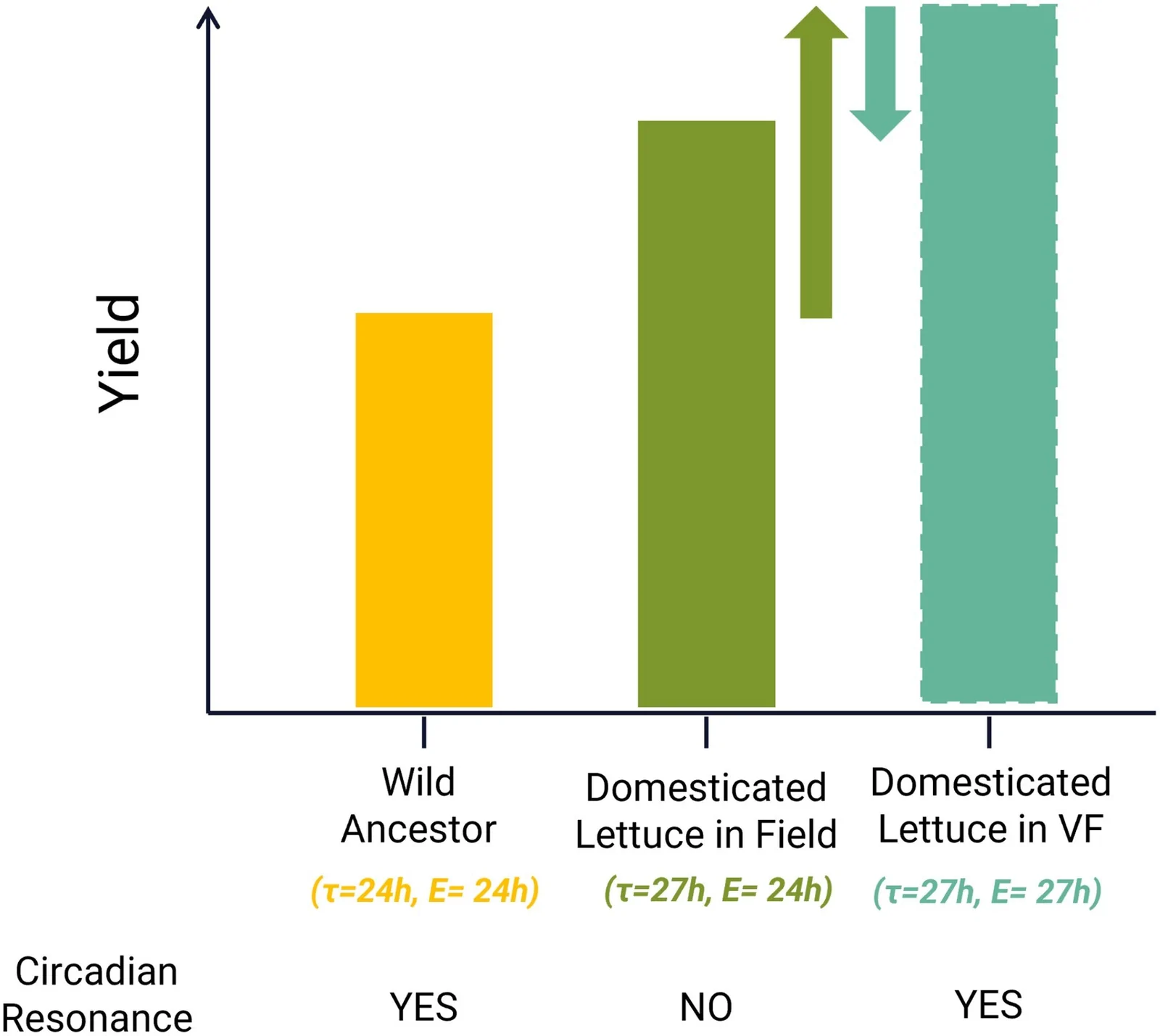
Hypothesis diagram illustrates the circadian resonance model in lettuce. If circadian resonance affects biomass accumulation, yield should be maximized when the external day length (E) matches the internal circadian period of the plant (tau, τ). Most cultivated lettuce accessions have an ∼27 h tau, while wild relatives and some cultivated accessions retain a 24 h rhythm. However, commercial lettuce is grown in the field or in greenhouses, under cycles of 24 h. Accordingly, most cultivated accessions are expected to perform best under 27 h day cycles, and wild accessions under 24 h cycles. These non-natural T27 cycles can only be implemented in controlled environments such as vertical farms (VFs), which allow precise manipulation of day-length and photoperiod. The arrows illustrate our predicted yield outcomes: the green upward arrow shows maximum yield potential of cultivated lettuce under resonant 27 h cycles, while the teal downward arrow shows the putative yield reduction caused by circadian dissonance under 24 h conditions. Current yields of lettuce plants grown under 24 h days represent maximum potential minus the potential dissonance penalty.

Here we investigate whether eliminating circadian misalignment through matched light–dark cycles can enhance lettuce yield under vertical farming conditions. We hypothesize that long-clock lettuce varieties (∼27 h) will exhibit superior performance under 27 h versus 24 h cycles, while normal-clock varieties (∼24 h) should show optimal performance under 24 h cycles (Fig. 1). We test this hypothesis using 20 lettuce accessions with previously characterized circadian periods (Anton-Sales *et al.*, 2025) across six light–dark treatments, evaluating biomass accumulation, quality metrics, and developmental timing. This study aims to establish whether circadian resonance represents a viable strategy for sustainable yield improvement in controlled-environment horticulture.

## Materials and methods

### Plant material

Eighteen *Lactuca* SSD (single seed descent) lines from the Centre for Genetic Resources in the Netherlands (CGN) were used, selected from a well-characterized public lettuce collection (http://www.wur.eu/cgnsc002) (Wei *et al.*, 2021). We included two modern commercial cultivars sourced from Rijk Zwaan, namely Rabello RZ and Frillice-Dunand, both indicated for CEA.

Circadian clock, bolting, budding, and flowering times were previously determined for CGN accessions (Anton-Sales *et al.*, 2025) and used to select genotypes with contrasting circadian clocks (25–28 h) and flowering times (88–158 d (Table 1). Seven accessions had 24 h clocks, nine had 27 h clocks, and two were wild relatives with 24 h clocks. Unless stated otherwise, only cultivated lettuce was used for statistical comparisons. The circadian and developmental parameters of commercial cultivars were unknown.

**Table 1.** Lettuce accessions used in this study

Accession	Flowering time (d)	Period (h)	Period nominal	Accession	Species	Population type	Country	Year	Name	Crop type	Cultivation
TKI-340	144	23	Normal (N)	US96UC23	*L. serriola*	Research line	NA	NA	*L. serriola*	NA	NA
TKI-003	95	23.9	Normal (N)	CGN04536	*L. sativa*	Cultivar	FRA	1847	Brune d’Hiver	Butterhead	Winter
TKI-013	88	24	Normal (N)	CGN04828	*L. sativa*	Cultivar	FRA	1842	Rougette du Midi a Graine Noire	Butterhead	Winter
TKI-011	102	24.12	Normal (N)	CGN04818	*L. sativa*	Cultivar	FRA	1902	Reine de Mai	Butterhead	Spring Summer Autumm
TKI-037	102	24.2	Normal (N)	CGN10910	*L. sativa*	NA	SRB	1972	Trgoviska	Butterhead	NA
TKI-051	102	24.4	Normal (N)	CGN13314	*L. sativa*	Cultivar	DEU	1957	Fruhlingsgruss	Butterhead	Spring
TKI-342	158	24.8	Normal (N)	CGN05271	*L. saligna*	Wild	SPA	NA	*L. saligna*	NA	NA
TKI-043	102	24.8	Normal (N)	CGN11340	*L. sativa*	Research line	NA	NA	UCDM14	Butterhead	Winter
TKI-062	95	25.2	Normal (N)	CGN19067	*L. sativa*	Cultivar	DEU	NA	Wintra	Butterhead	Winter
TKI-054	123	26.8	Long (L)	CGN14635	*L. sativa*	Cultivar	NLD	1984	Dabora	Butterhead	Summer
TKI-024	111	26.9	Long (L)	CGN05257	*L. sativa*	Cultivar	NLD	1914	Wonder van Voorburg	Butterhead	Autumm
TKI-495	111	27	Long (L)	PV15020	*L. sativa*	Cultivar	NLD	1972	Olof	Butterhead	NA
TKI-034	116	27.1	Long (L)	CGN09340	*L. sativa*	Cultivar	URY	NA	Semi-Morada	Butterhead	NA
TKI-021	130	27.1	Long (L)	CGN05175	*L. sativa*	Cultivar	FRA	1900	Blonde du Cazard race Reine de Juillet	Butterhead	Summer
TKI-056	116	27.8	Long (L)	CGN14681	*L. sativa*	Cultivar	FRA	1985	Perlane	Butterhead	NA
TKI-019	111	27.9	Long (L)	CGN05140	*L. sativa*	Cultivar	FRA	1975	Capitan	Butterhead	Outdoor
TKI-004	116	28.3	Long (L)	CGN04574	*L. sativa*	Cultivar	FRA	1926	Du Bon Jardinier	Butterhead	NA
TKI-047	111	29	Long (L)	CGN11379	*L. sativa*	Cultivar	NLD	1976	Virex	Butterhead	NA
AF-001	NA	NA	Unknown (U)	41-CO7708 RZ	*L. sativa*	Cultivar	NLD	2021	Rabello	Cos Romaine	Indoor
AF-002	NA	NA	Unknown (U)	44–22 RZ	*L. sativa*	Cultivar	NLD	2019	Dunand	Frillice type	Outdoor

A total of 20 accessions were selected, including 18 genotypes for which we previously characterized the circadian clock and flowering time (Anton-Sales *et al*., 2025). These accessions were chosen to represent different circadian clock and bolting/flowering times for testing the circadian resonance hypothesis. Two wild lettuce accessions (*Lactuca serriola*, TKI-340 and *L. saligna*, TKI-342) and two commercial cultivars (denoted as R and F) were included. The year indicates the collection date for CGN material (when known) (Wei *et al*., 2021) or the market release year for commercial cultivars.

### Plant growth conditions

Lettuce performance trials were conducted at Avery Farms, a commercial vertical farm in British Columbia, Canada. Experimental accessions (Table 1) were sown into polyurethane sponges in trays (300 plants per tray). Seeds were stratified at 4 °C for 4 d in darkness, then transferred to growth chambers where seedlings remained covered for 2 d before exposure to 5000 K LED lighting for 6 diels. At 10 diels, plants were transplanted into hydroponic grow racks. The setup consisted of two vertical racks with five horizontal levels holding 384 plants per level (Supplementary Fig. S1) (1920 plants per rack). Plants grew on floating Styrofoam rafts with recirculating nutrient solution (electrical conductivity 1.5 mS cm^–1^, pH 6.7) containing commercial hydroponic fertilizer and calcium nitrate. Plants were grown at 48 plants m^–2^ until 30 diels; those continuing to 46 diels were thinned to 24 plants m^–2^ at 32 diels. Plants were grown under 4000 K LED lighting at 250 µmol m^–2^ s^–1^ or 200 µmol m^–2^ s^–1^ depending on treatment (Table 2). Treatments were assigned to individual levels and separated by light-blocking curtains. Plant positions were randomized using a row–column design (v1.5.0) (Murillo and Gezan, 2026) to minimize edge effects.

**Table 2.** Light recipes used in each of the treatments tested in our experiments at Avery Farms

Day length (h)	Photoperiod (Light:dark)	DAS	Diels	Total light (h)	Light intensity (PPFD)	DLI (mol m^−2^ d^−1^)	Cumulative light (mol m^−2^)
24	16:8	30	30	480	250	14.4	432
24	20:4	30	30	600	250	18*^a^*	540
24	20:4	30	30	600	200*^b^*	14.4	432
27	20:7	34	30	600	250	18*^a^*	540
27	20:7	34	30	600	200*^b^*	14.4	432
27	16:11	34	30	480	250	14.4	432
24	16:8	46	46	720	250	14.4	662.4
24	20:4	46	46	900	250	18*^a^*	828
24	20:4	46	46	900	200*^b^*	14.4	662.4
27	16:11	51.75	46	720	250	14.4	648
27	20:7	51.75	46	900	250	18*^a^*	810
27	20:7	51.75	46	900	200*^b^*	14.4	648

Twelve harvest points were recorded following 30 and 46 diel cycles under six distinct treatments combining two day-lengths (24 h and 27 h) with three photoperiod/light intensity regimes (16 h light/8 h or 11 h dark at 250 μmol m^−2^ s^−1^, 20 h light/4 h or 7 h dark at 250 μmol m^−2^ s^−1^, and 20 h light/4 h or 7 h dark at 200 μmol m^−2^ s^−1^). The table summarizes the number of 24 h days after sowing (DAS), number of diel cycles (light/dark units), total hours of light exposure, light intensity (PPFD), daily light integral (DLI), and cumulative light received.

^*a*^High-DLI treatments (18 mol m^−2^ d^−1^), where the photoperiod was extended to 20 h without reducing light intensity, resulting in a higher DLI than standard conditions.

^*b*^Lower light intensity treatments, where PPFD was reduced from 250 μmol m^−2^ s^−1^ to 200 μmol m^−2^ s^−1^ to match the DLI of standard cultivation conditions (16:8 at 250 μmol m^−2^ s^−1^, DLI=14.4 mol m^−2^ d^−1^), despite the extended 20 h photoperiod (20:4 or 20:7 at 200 μmol m^−2^ s^−1^, DLI=14.4 mol m^−2^ d^−1^).

Temperature was 22 °C day/19 °C night. Light intensity was measured at plant height using a LI-250A PAR meter (Li-Cor Biosciences, Lincoln, NE, USA). Relative humidity was 65–75%, wind speed ∼0.6 mph during light hours, and CO_2_ remained ambient.

### Treatments

**Fig. 2.**
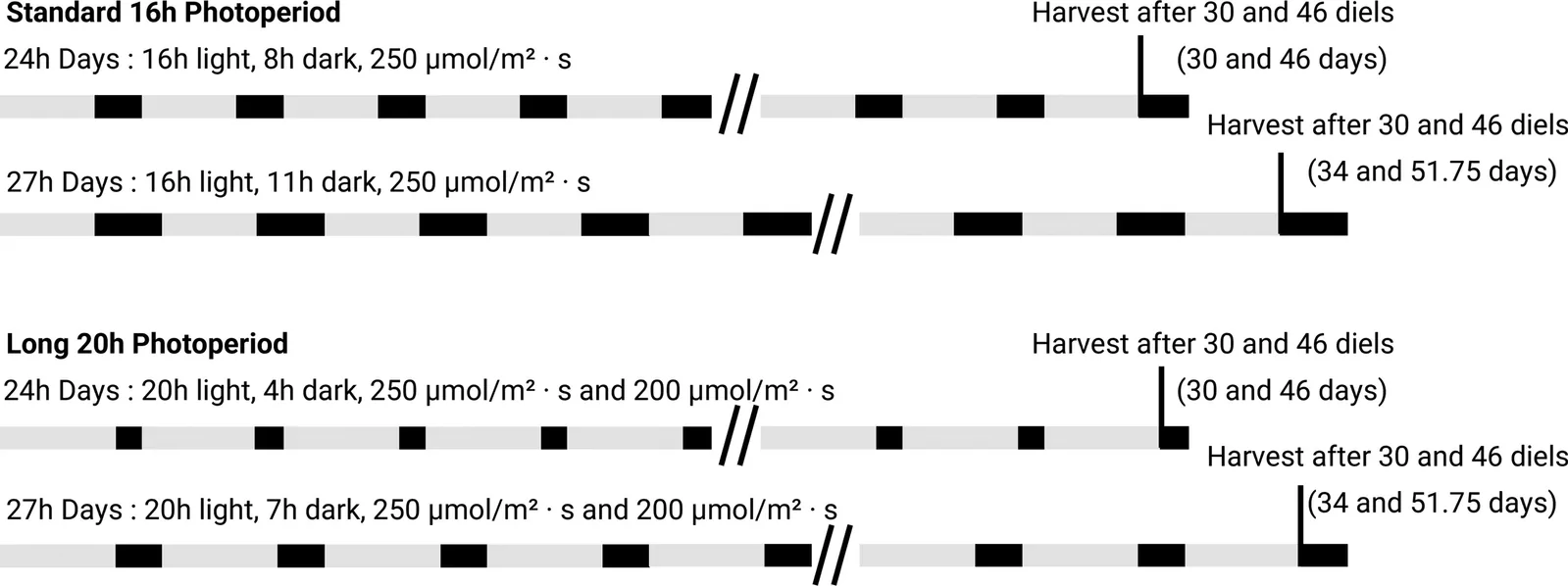
Experimental conditions tested in the Avery Farms trials. Lettuce accessions were grown under two day-lengths—24 h and 27 h cycles—with two photoperiod treatments: a standard 16 h light/8 h dark (16:8 for 24 h; 16:11 for 27 h) and an extended 20 h light/4 h dark (20:4 for 24 h; 20:7 for 27 h). Light intensity was set at 250 µmol m^–2^ s^–1^, except for an additional treatment where the 20 h photoperiod used 200 µmol m^–2^ s^–1^ to match the daily light integral (DLI) of the 16 h treatment. Harvests were performed at 30 and 46 diel cycles (day–night units), as well as at the equivalent time points in human-standard 24 h days (34 d and 51.75 d for T27) so plants received the same number of light hours in the 24 h and 27 h treatments.

Plants grew under 24 h (T24) or 27 h (T27) diel cycles with two photoperiod treatments: 16:8 and 20:4 (light:dark hours) under T24, and 16:11 and 20:7 under T27 (Fig. 2). All 16 h photoperiod treatments used 250 µmol m^−2^ s^−1^, yielding daily light integral (DLI)=14.4 mol m^−2^ d^−1^. The 20 h treatments at 250 µmol m^−2^ s^−1^ yielded DLI=18 mol m^−2^ d^−1^. To isolate photoperiod effects from light dose, additional treatments (20:4 lowI and 20:7 lowI) used 200 µmol m^−2^ s^−1^, matching the 16 h DLI (14.4 mol m^−2^ d^–1^) (Table 2).

Plants were harvested at 30 and 46 diels (equivalent developmental stages under T27). At harvest, treatments with identical DLI had received equal cumulative light and total light hours, incurring no additional lighting costs. However, as each T27 diel is 12.5% longer in calendar time than T24 (27/24 h), the T27 crop cycles required 12.5% more real time to reach the equivalent harvest points (33.75 d and 51.75 d versus 30 d and 46 d, respectively).

### Phenotyping

All phenotypic observations are provided in Supplementary Table S1.

#### Fresh weight

Harvested plants were cut above the substrate and weighed (Kern PCR 6000) after 30 and 46 diels (*n*≥7–16 per accession/treatment).

#### Dry weight and dry matter

Plants (*n*=3–8) were dried for 72 h at 105 °C in industrial ovens. Dry matter was calculated as DW/FW×100.

#### Imaging

Top-down images of 3–5 plants per accession were captured at harvest with reference rulers for calibration and under a black background.

#### Tipburn

**Fig. 3.**
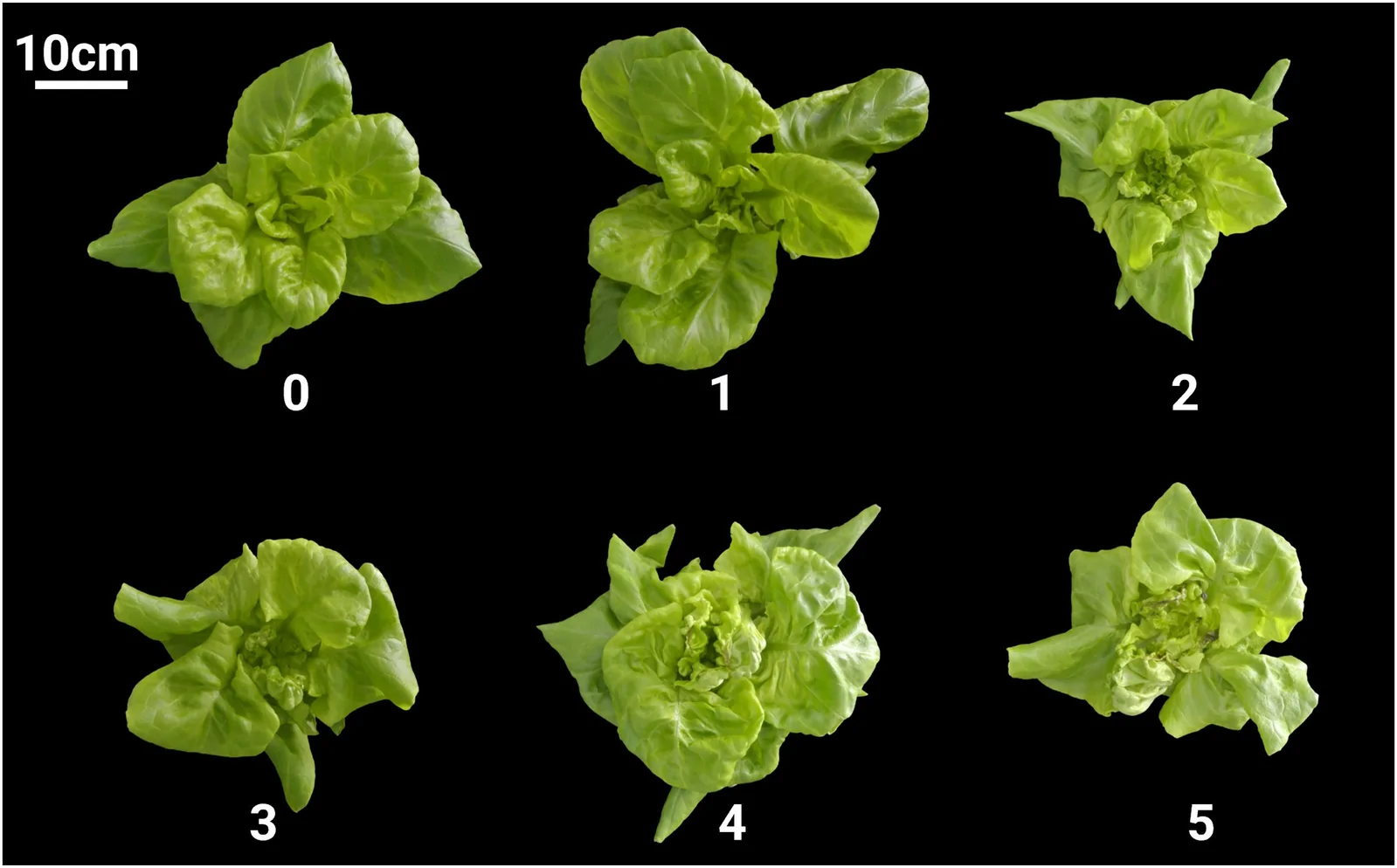
Visual tipburn severity scale used for lettuce phenotyping. Representative photographs of lettuce plants illustrating the six-point tipburn scoring scale, ranging from 0 (no tipburn symptoms) to 5 (severe tipburn with extensive necrosis of inner leaves). Score 0 indicates healthy plants with no visible damage; scores 1–2 reflect early-stage symptoms with minor marginal browning on young leaves; scores 3–5 represent progressive necrosis affecting an increasing proportion of the inner canopy and considered unmarketable (severe tipburn). Scale bar=10 cm.

Tipburn was assessed with a visual scoring (0–5 scale: 0=no, 5=highest incidence, Fig. 3).

#### Bolting

Bolting was assessed using binary scoring (0=no bolting, 1=bolting initiation). Bolted plants were photographed.

### Statistical analysis

Analyses used R 4.4.0 and Python 3.11.5. Normality and variance homogeneity were tested using Shapiro–Wilk and Levene’s tests. When assumptions were met (*P*>0.05), we fit lm (FW ∼ Treatment); when violated, we used lm [rank(FW) ∼ Treatment]. Post-hoc comparisons used estimated marginal means (Searle *et al.*, 1980) via the emmeans package (1.11.1) with false discovery rate (FDR)-adjusted *P*-values. For pairwise comparisons, we used *t*-tests or Mann–Whitney U-tests if assumptions were violated. Categorical variables were analysed using χ^2^ or Fisher’s exact tests when counts were small (*n*≤3).

Additionally, we examined data from treatments with an equivalent DLI (14.4 mol m^−2^ d^−1^) using a linear mixed-effects model. Because noctoperiod length is nested within diel cycles (8 h and 4 h nights occur only under 24 h cycles; 11 h and 7 h nights occur only under 27 h cycles), including both photoperiod and noctoperiod as separate terms would create perfect collinearity (photoperiod+noctoperiod=diel). To avoid this while testing biologically meaningful effects, we modelled photoperiod as an interaction with diel:


FW∼Clock×Diel+Diel×Photoperiod+(1|Accession)

This model tests whether: (i) the benefit of 27 h cycles depends on circadian clock type (Clock×Diel interaction, our primary hypothesis), and (ii) the effect of photoperiod length differs between 24 h and 27 h cycles (Diel×Photoperiod interaction). Within each diel cycle, the Diel×Photoperiod interaction compares 16 h versus 20 h photoperiods, which correspond to 8 h versus 4 h nights (24 h cycles) and 11 h versus 7 h nights (27 h cycles).

Maximum likelihood estimation (REML=FALSE) was used for model fitting to enable likelihood ratio tests for model comparison. Model complexity was evaluated using likelihood ratio tests and the Akaike information criterion (AIC). Type III ANOVA assessed fixed effects using Satterthwaite’s method for degrees of freedom approximation. Marginal *R*^2^ (variance explained by fixed effects) and conditional *R*^2^ (variance explained by fixed and random effects) were calculated using the performance R package (version 0.12.4).

## Results

### Circadian resonance drives yield increases without additional light hours in long-clock lettuce

**Fig. 4.**
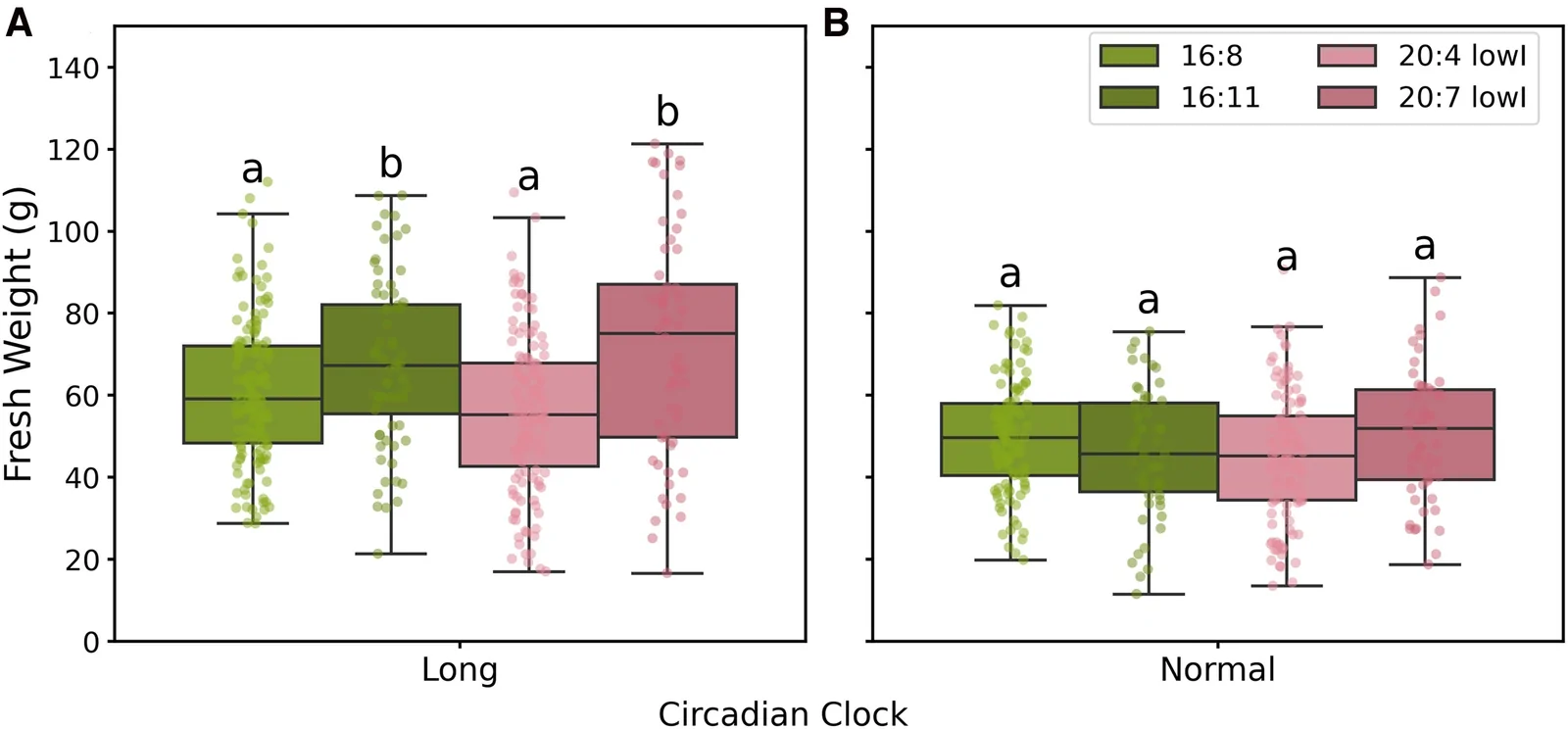
FW accumulation after 30 diels of cultivated lettuce accessions with long (∼27 h) (A) and normal (∼24 h) (B) circadian clocks under different photoperiod and day-length treatments. Plants were grown under 16 h light/8 h dark (16:8, green), 16 h light/11 h dark (16:11, dark green), and with a reduced light intensity with 20 h light/4 h dark (20:4lowI, light rose), and 20 h light/7 h dark (20:7, lowI rose). FW was measured after 30 d (24 h) or the equivalent in 27 h time (34 d of 24 h). Statistical comparisons were performed separately within each clock group [Group 1: long clock (A), Group 2: normal clock (B)] using a rank-transformed linear model, followed by estimated marginal means and FDR-adjusted pairwise comparisons. Letters indicate statistically significant differences among treatments (*P*<0.05); treatments sharing a letter are not significantly different. Source data are given in Supplementary Table S1, and statistical data in Supplementary Table S3.

In our study, we grew >2000 lettuce heads across six experimental treatments to quantify whether circadian resonance influences biomass accumulation and crop quality. We included seven accessions with standard 24 h internal clocks, nine accessions with 27 h circadian clocks, two commercial varieties currently in cultivation with unknown circadian periods, and two wild lettuce accessions with normal clocks. Consistently long-clock accessions accumulated more biomass than those with standard 24 h clocks (Supplementary Fig. S2), and this inherent advantage was even more marked when long-clock lines were cultivated under matching 27 h environmental cycles (Fig. 4A).

We designed treatments that aligned internal rhythms with external cycles while holding total light energy constant, using the industry standard of 16 h of light followed by 8 h darkness at 250 μmol m^−2^ s^−1^ (DLI=14.4) as a reference. In four of our treatments, we maintained identical DLIs of 14.4 mol m^−2^ d^−1^, achieved by redistributing the same photon flux across different photoperiod lengths. This approach ensured that yield differences reflected circadian alignment rather than increased energy input.

Under standard cultivation conditions (16 h photoperiod at 250 μmol m^−2^ s^−1^), long-clock accessions grown under resonant 27 h cycles (16:11 light:dark) accumulated 11.2% more biomass than the same genotypes under non-resonant 24 h cycles (16:8), averaging 68 g versus 61 g, respectively (Fig. 4A). This yield increase under resonant conditions intensified when photoperiods were extended to 20 h: plants under 27 h cycles (20:7 lowI) yielded 72 g compared with 56 g under 24 h cycles (20:4 lowI), representing a 29% increase.

Importantly, these substantial yield gains required no additional light energy. We simply extended dark periods: from 8 h to 11 h (16:8 to 16:11) or from 4 h to 7 h (20:4 lowI to 20:7 lowI), while harvesting after equivalent numbers of light–dark cycles. Moreover, these productivity improvements during the 27 h treatments came without quality trade-offs, as overall visual quality (OVQ) remained comparable with the standard 16:8 (Supplementary Fig. S3).

In stark contrast, normal-clock accessions (24 h internal rhythms) showed minimal response to cycle length variation, displaying only slight, non-significant differences between 24 h and 27 h treatments (Fig. 4B).

### Circadian resonance maintains annual productivity under extended diel cycles in long-clock lettuce accessions

To assess whether the longer cultivation period associated with 27 h diel cycles reduces annual productivity, we normalized FW by elapsed days and projected yield per m^2^, accounting for rotation frequency (12.2 cycles year^−1^ versus 10.8 cycles year^−1^ for 24 h and 27 h cycles, respectively; Supplementary Fig. S4). Daily biomass accumulation rates remained higher in long-clock accessions under 27 h cycles compared with normal-clock accessions under any condition (Supplementary Fig. S4B). Projected annual yield per m^2^ was equivalent or greater for long-clock accessions under 27 h cycles relative to the same accessions grown under 24 h cycles (Supplementary Fig. S4C, D), demonstrating that the 11% reduction in annual rotation frequency is fully offset by the biomass gained through circadian resonance. No such compensation was observed in normal-clock accessions, for which 27 h cycles reduced both daily growth rate and projected annual yield (Supplementary Fig. S4B–D).

### Statistical modelling confirms circadian resonance as the primary driver

To test factor interactions, we developed a mixed-effects model analysing treatments with equivalent DLI (16:8, 16:11, 20:4 lowI, 20:7 lowI). The model incorporated clock genotype (normal or long), diel cycle length (24 h versus 27 h), and their interaction as fixed effects, with random intercepts for accession. Noctoperiod length is nested within diel cycles (4 h and 8 h nights occur only in 24 h cycles; 7 h and 11 h nights occur only in 27 h cycles), so we included photoperiod as an interaction with diel rather than as a separate main effect to avoid collinearity. Model selection via likelihood ratio tests confirmed that including the Clock×Diel interaction and Diel×Photoperiod interaction significantly improved fit over main effects alone (ΔAIC=25.8, χ^2^=29.81, *P*<0.001; Supplementary Fig. S5).

**Fig. 5.**
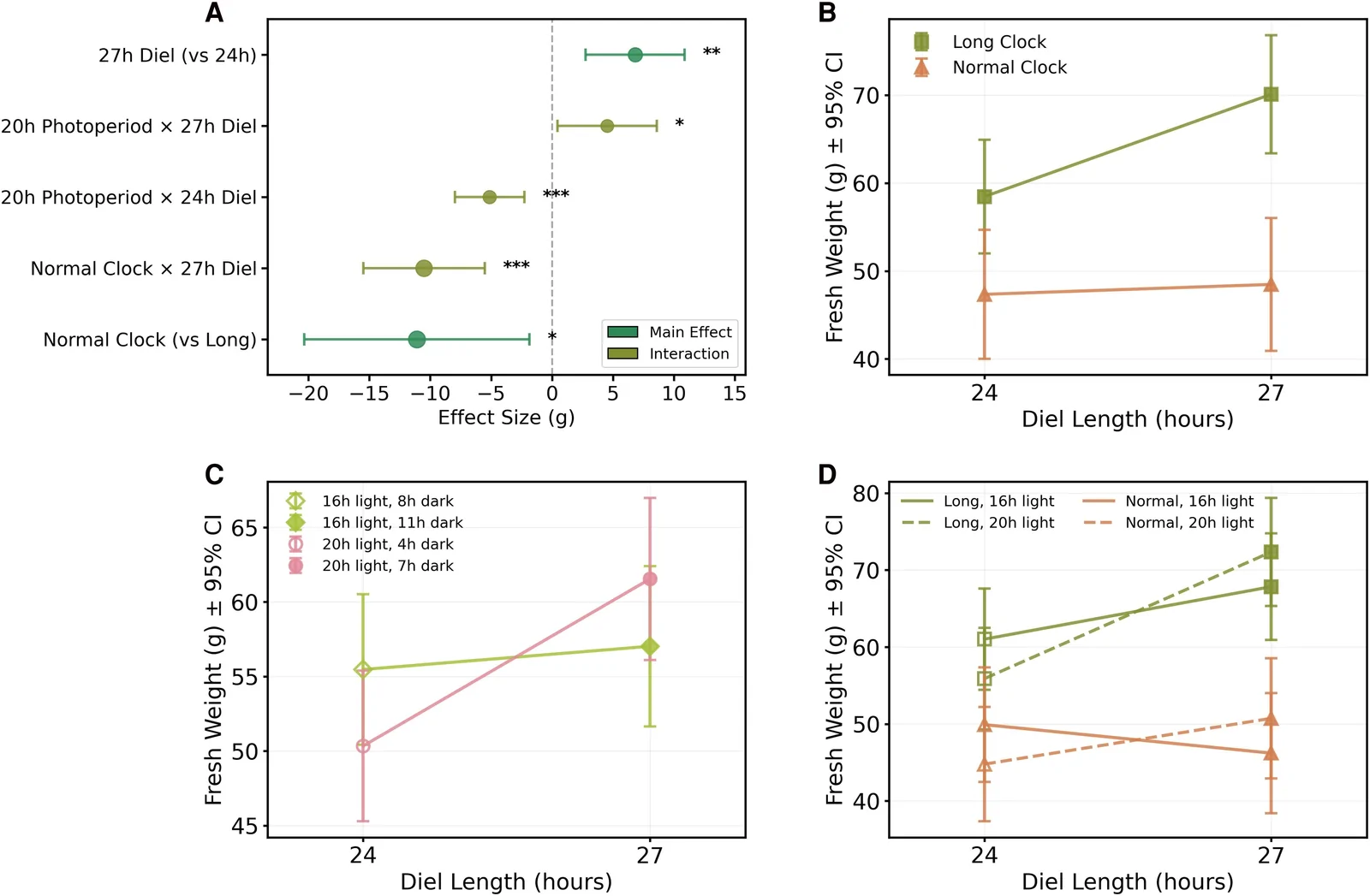
Comprehensive analysis of circadian clock genotype effects on plant FW under different photoperiodic and diel conditions and with the same daily light integral (DLI) of 14.4 mol m^−2^ d^−1^. (A) Forest plot showing fixed effects from mixed-effects model analysis with 95% confidence intervals. The model includes clock genotype (normal versus long), diel cycle length (24 h versus 27 h), their interaction (Clock×Diel), and the Diel×Photoperiod interaction. Asterisks indicate significance levels (***P<0.001, **P<0.01, *P<0.05). (B) Clock×Diel interaction showing that the long-clock genotype (squares) FW increases dramatically under extended (27 h) compared with standard (24 h) diel cycles, while the normal-clock genotype (triangles) shows minimal response. (C) Diel×Photoperiod interaction demonstrates that optimal photoperiod length depends on cycle duration. Diamonds represent 16 h photoperiods and circles represent 20 h photoperiods. Open symbols indicate shorter night periods (8 h for 16 h photoperiod, 4 h for 20 h photoperiod), while filled symbols indicate longer night periods (11 h for 16 h photoperiod, 7 h for 20 h photoperiod). Under 24 h cycles, 16 h photoperiods are superior, while under 27 h cycles, 20 h photoperiods are better in all accessions tested pooled together. (D) Three-way interaction (Clock×Diel×Photoperiod) showing all treatment combinations. Squares represent long clock, and triangles represent normal clock. Solid lines indicate 16 h photoperiods and dashed lines indicate 20 h photoperiods. Open symbols show shorter night periods (8 or 4 and 7 h) while filled symbols show longer night periods. The divergent responses between clock types under different diel lengths illustrate that circadian resonance effects depend on both internal clock period and photoperiod regime.

The final model explained 35.4% of total variance (conditional *R*^2^; Supplementary Fig. S5), with significant effects from clock type (*F*_1_,_16_=14.32, *P*=0.002) and the Diel×Photoperiod interaction (*F*_2_,_716_=8.61, *P*<0.001) (Fig. 5A; Supplementary Table S2). Critically, the Clock×Diel interaction showed the largest effect (*F*_1_,_716_=17.08, *P*<0.001), demonstrating that the benefit of 27 h cycles depends on circadian clock type and providing direct evidence for circadian resonance.

Under standard 24 h cycles, long-clock plants showed an 11.1 g biomass advantage over normal-clock plants. However, under resonant 27 h cycles, this advantage nearly doubled to 21.6 g, representing a 94% increase in the long-clock benefit (Fig. 5B; Supplementary Table S2). This marked enhancement under resonant conditions provides statistical evidence that circadian alignment drives yield improvements.

Moreover, estimated marginal means revealed that optimal light–dark periods depend on diel length (Fig. 5C). Under 24 h cycles, 16 h photoperiods yielded 5.1 g more than 20 h photoperiods (*P*<0.001), while under 27 h cycles, 20 h photoperiods were superior by 4.5 g (*P*=0.030; Supplementary Table S2). Thus, there is an optimal noctoperiod within each diel. The optimal treatment combination was long-clock genotype with 27 h cycles and 20 h photoperiod (7 h darkness), achieving 72.4 g. This results in a 62% improvement over the worst-performing combination: normal-clock genotype with 24 h cycles and 20 h photoperiod (4 h darkness; Supplementary Table S2).

### High-energy treatments maximize biomass but compromise quality

To test the limits of yield increase, we grew the plants in two more treatments, where we extended photoperiods to 20 h as before (20:4 and 20:7) under 24 h and 27 h diel lengths but maintaining standard light intensity (250 μmol m^−2^ s^−1^). Thus, we created high-DLI treatments (18 mol m^−2^ d^−1^). Both long-clock and normal-clock varieties achieved maximum biomass accumulation (Table 3; Supplementary Figs S6–S9) under these treatments (Supplementary Tables S3, S4). Again, long-clock plants outperformed normal-clock varieties; however, notably, cycle length (24 h versus 27 h) made no significant difference under the high-energy treatments, with circadian resonance effects disappearing when a high light input was provided (Supplementary Fig. S10).

**Table 3.** Summary of FW mean values for all accessions with a long (∼27 h) and normal (24 h) circadian clock under all treatments tested after 30 growth cycles

Treatment	Long clock FW (g)	Normal clock FW (g)
16:8	61.00	49.80
16:11	67.83	46.08
20:4 (low I)	55.96	45.10
20:7 (low I)	72.13	50.71
20:4	77.16	55.69
20:7	78.95	55.71

Statistical comparisons are summarized in Supplementary Fig. S7.

However, the maximum yields under the high-DLI treatments (20:4 and 20:7) came with substantial quality costs. Plants exposed to these treatments exhibited visible morphological aberrations including darker, thicker, curlier leaves with irregular rosette structure compared with standard 16:8 morphology (Supplementary Figs S11, 12). The most severe aberrations occurred under 20:4 treatment, which provided the shortest night period (4 h, Supplementary Fig. S11). Additionally, these high-energy treatments triggered increased stress symptoms including elevated tipburn incidence (Supplementary Fig. S13), rendering many plants unmarketable despite higher biomass.

### Quality maintenance under resonant conditions

**Fig. 6.**
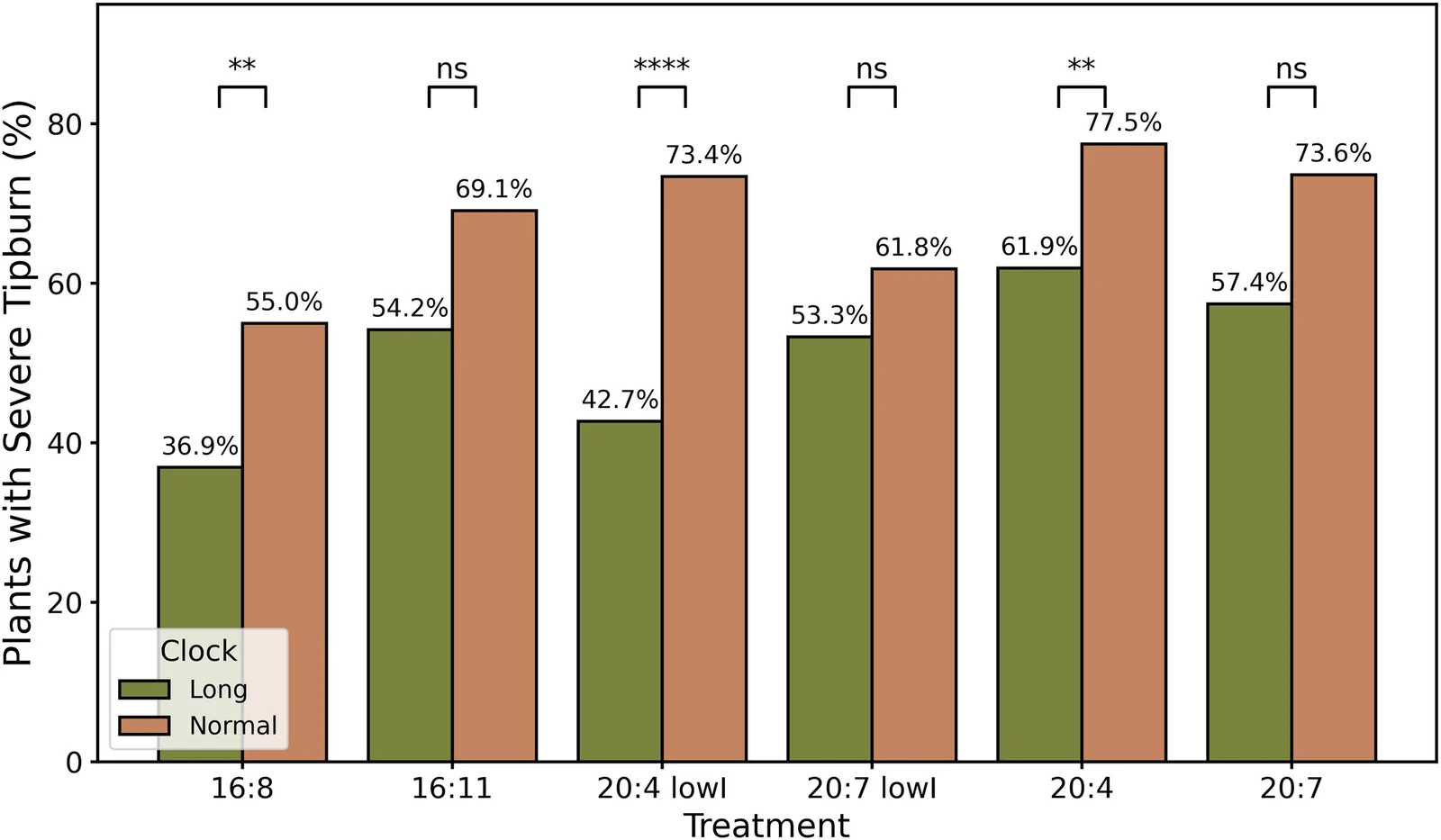
Percentage of plants exhibiting severe tipburn (score ≥3) symptoms by circadian clock type under all tested treatments. Accessions with long (∼27 h; olive) circadian clocks showed lower severe tipburn incidence than those with normal (∼24 h; orange) clocks. Significance brackets indicate pairwise Fisher’s exact tests between clock types within each treatment (**P<0.01, ****P<0.0001, ns: not significant).

Long-clock accessions demonstrated significantly superior tipburn resistance compared with normal-clock varieties across all treatments. Overall, 49.8% of long-clock plants showed severe tipburn (score ≥3) versus 68.5% in normal-clock varieties (χ^2^=38.56, *P*<0.0001; Fig. 6).

Tipburn severity varied by accession and treatment (Supplementary Fig. S13), with the highest incidence occurring under high-energy conditions (20:4 and 20:7). This pattern reflects the fundamental trade-off between rapid growth and quality. However, long-clock plant yield increases under circadian-resonant conditions were also achieved without significantly higher tipburn incidence (Supplementary Fig. S13).

### Circadian resonance restores bolting timing

Long-clock lettuce varieties consistently exhibit delayed bolting under field and greenhouse conditions (24 h days) compared with normal-clock varieties (Anton-Sales *et al.*, 2025), a trait considered a direct consequence of domestication. Our results reveal that this developmental delay may result from chronic circadian misalignment rather than inherent developmental reprogramming.

After 30 diel cycles, this pattern held: long-clock plants showed minimal bolting under most conditions (<2% under standard photoperiods and low-intensity treatments; Supplementary Figs S14, S15A, C), while normal-clock plants exhibited substantially higher bolting rates of 7–40% (Supplementary Figs S14, S15A, C), confirming the delayed bolting phenotype previously observed in field trials. The highest bolting incidence in long-clock plants was found under the 20:4 treatment.

**Fig. 7.**
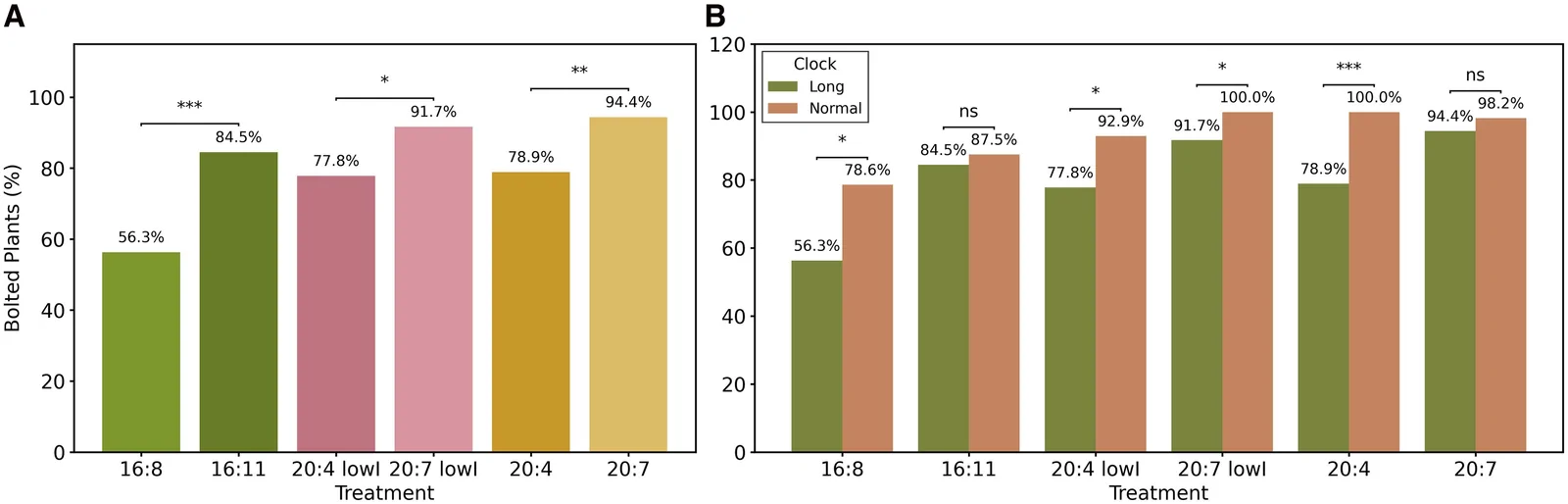
Circadian resonance restores natural bolting timing in long-clock lettuce. (A) Bolting incidence in long-clock accessions (∼27 h) at 46 diel cycles across six photoperiod and cycle length treatments: 16:8, 16:11, 20:4 lowI, 20:7 lowI, 20:4, and 20:7. Bars represent the percentage of plants that initiated bolting. (B) Comparison of bolting frequency between long-clock (olive) and normal-clock (orange) accessions under all six treatments at 46 diel cycles. Bars represent the percentage of bolted plants. Asterisks indicate statistically significant differences between clock types within each treatment (Fisher’s exact test: *P<0.05, **P<0.01, ***P<0.001; ns=not significant).

However, by 46 diel cycles, resonant conditions (27 h days) eliminated this difference. Significantly more long-clock accessions bolted under all 27 h treatments compared with 24 h treatments, reversing the delayed developmental pattern characteristic of field-grown plants (Fig. 7A). Under 16:11 and 20:7 treatments, long-clock varieties achieved bolting frequencies comparable with normal-clock varieties (Fig. 7B), indicating restoration of natural reproductive timing when internal and external rhythms synchronize. Importantly, this restoration occurred after 45 d, far beyond the cultivation window (30 d).

### Commercial cultivar responses

**Fig. 8.**
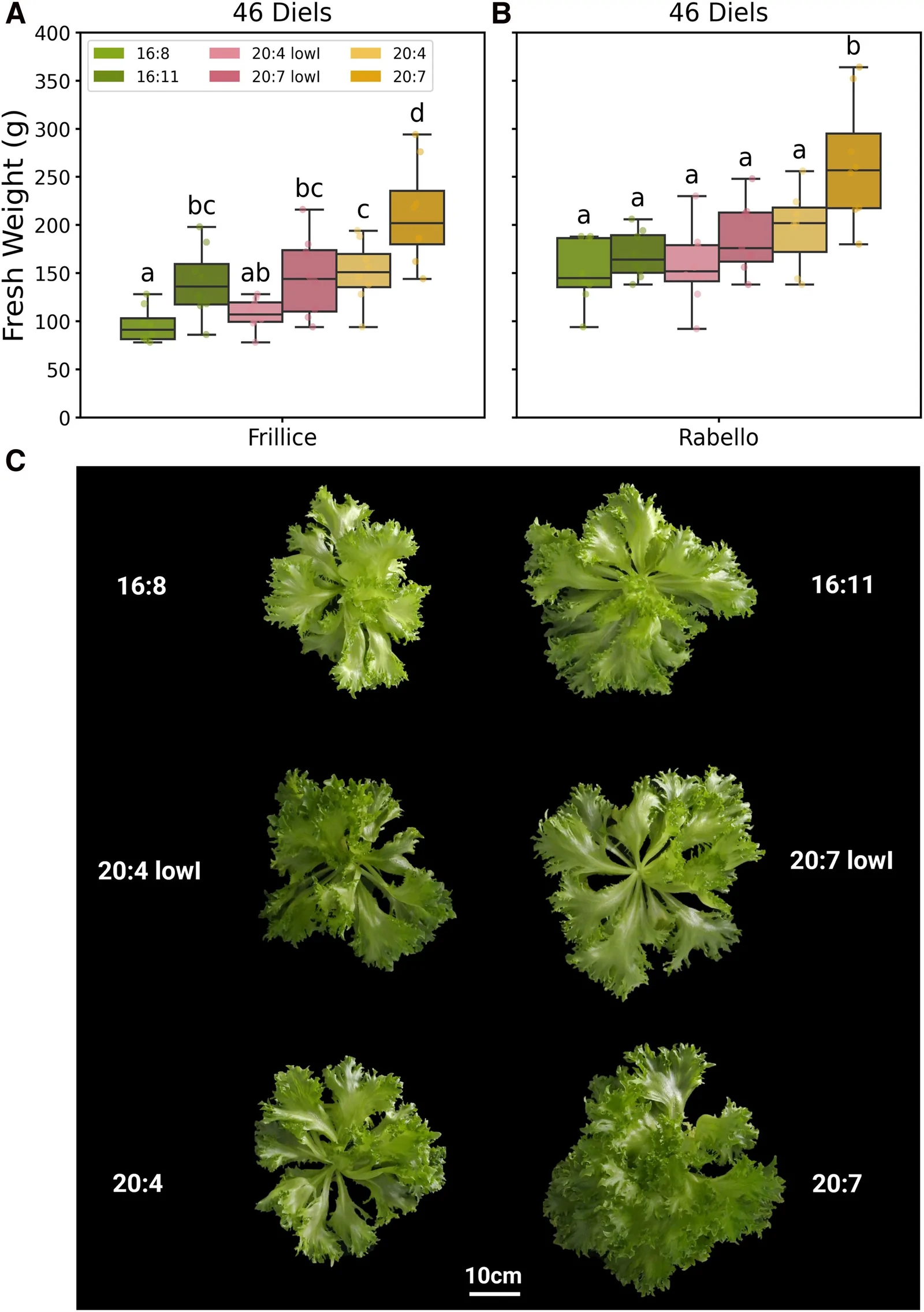
Commercial cultivar Frillice exhibits strong yield increase under 27 h days at commercial harvest time point. FW of Frillice (A) and Rabello (B) at 46 diel cycles under six treatments combining 24 h and 27 h cycles with different photoperiods. Frillice shows substantial yield increases under 27 h cycles: +46.7% (16:11 versus 16:8), +35.6% (20:7 lowI versus 20:4 lowI), and +39.7% (20:7 versus 20:4). Rabello demonstrates yield stability across treatments. Treatments with ‘lowI’ used reduced light intensity (200 μmol m^−2^ s^−1^) to match DLI with 16 h treatments (14.4 mol m^−2^ d^−1^); standard 20 h treatments provided higher DLI (18 mol m^−2^ d^−1^). Box plots show median, quartiles, and individual points. Letters indicate significant differences within cultivars (*P*<0.05). (C) Representative images of Frillice showing larger rosettes under T27 conditions (16:11, 20:7 lowI, 20:7) compared with 24 h cycles (16:8, 20:4 lowI, 20:4) while maintaining quality. Scale bar=10 cm.

In our study, we included two commercial cultivars, Frillice and Rabello, with unknown clock genotypes. Frillice, a cultivar optimized for controlled-environment production with a typical 46 d growing cycle, exhibited strong responses to our treatments. Under 27 h treatments, Frillice achieved substantial yield increases: 16:11 produced 140.5 g versus 95.8 g under 16:8 (+46.7% increase), 20:7 low-intensity yielded 145.8 g versus 107.5 g under 20:4 low-intensity (+35.6% increase), and the high-energy 20:7 treatment reached 211 g versus 151 g under 20:4 (+39.7% increase) (Fig. 8A). Thus, Frillice highly benefits from the T27 treatments, while maintaining excellent morphological quality, even under the high-DLI 20:7 treatment (Fig. 8C).

At the earlier 30 diel time point, Frillice showed no significant yield effects, with biomass remaining below 40 g across all treatments (Supplementary Fig. S16A). This response indicates that biomass benefits might accumulate over time, becoming economically significant only at commercial harvest maturity.

On the other hand, Rabello, a Cos-type cultivar, demonstrated remarkable yield stability across treatments, with consistent performance regardless of cycle length except under the highest energy treatment (20:7), where bolting was also highest (Fig. 8B; Supplementary Fig. S15). This commercial cultivar achieved maximum biomass under 20:7 (265 g) but with considerable morphological variation (Supplementary Fig. S17), and best quality under the standard 16:8 treatment.

Finally, both commercial cultivars showed the highest overall quality, with consistently low tipburn scores (≤2) across all treatments regardless of photoperiod or cycle length (Supplementary Fig. S13), and delayed bolting compared with gene bank accessions (Supplementary Fig. S15). However, in line with a putative resonant effect, Frillice exhibited accelerated bolting under 27 h treatments at 46 diels, with >60% bolting under 20:7 low-intensity and ∼40% under 16:11 and 20:7, again indicating a putative restoration of natural developmental timing under resonant conditions.

## Discussion

### Circadian resonance as a transformative horticultural strategy

This study provides the first demonstration that circadian resonance, the alignment of internal biological rhythms with environmental cycles, can deliver substantial yield improvements (11–29%) in a crop species and under commercial growing conditions. By matching 27 h environmental cycles to the internal clocks of long-clock lettuce varieties, we achieved significant biomass gains through biological optimization rather than increased light input.

Importantly, these yield increases were accomplished at equivalent lighting energy (Table 2). Plants grown under 27 h cycles received the same total light hours as those under 24 h cycles but accumulated significantly more biomass (Fig. 4A). While completion of 30 cycles of 27 h requires more calendar time (12.5% more) than 30 cycles of 24 h, incurring additional climate control costs, lighting still represents 60–80% of operational expenses in vertical farms and night hours incur modest costs (Kozai *et al.*, 2019). In our study, we did not use any additional light input, and indeed, light schedules were naturally shifted daily, in a way that many lighting hours naturally fall during normal-world night hours, when electricity prices drop. While this requires careful operational planning, the potential for significant yield gains without light cost increases represents an innovative cultivation strategy for indoor agriculture.

Furthermore, despite completing fewer annual rotations under 27 h cycles, projected yield per unit growing area remained equivalent or greater in long clock-accessions compared with the same genotypes grown under 24 h cycles (Supplementary Fig. S4). This confirms that the reduction in rotation frequency when cultivating under 27 h days is fully offset by the biomass gained per cycle.

### Extended dark periods coupled with circadian resonance drive yield in long-clock lettuce

When comparing our treatments with identical DLIs (14.4 mol m^−2^ d^−1^), we revealed that the primary driver of yield improvement in long-clock lettuce was 27 h diel cycles (Fig. 5A). Specifically, within long-clock accessions, both 27 h treatments (16:11 and 20:7 lowI) significantly outperformed their equivalent 24 h treatments (16:8 and 20:4 lowI), with no significant photoperiod effect within each diel (T24, T27) (Fig. 4A). This demonstrates that matching the environmental cycle to the circadian clock period (circadian resonance) is the key determinant of yield improvements found in our study.

When photoperiod was held constant and diel length was extended from 24 h to 27 h, biomass increased substantially: 16:11 outperformed 16:8 by 11%, and 20:7 lowI outperformed 20:4 lowI by 29%. This improvement necessarily involves both circadian resonance and extended noctoperiods, as extending the diel cycle from 24 h to 27 h while maintaining photoperiod inherently adds 3 h of darkness. Our model backed up these observations, showing that the Clock×Diel interaction had the largest effect size on yield (*F*_1_,_716_=17.08, *P*<0.001), confirming the effects of circadian resonance (Supplementary Fig. S5B; Supplementary Table S2). The Diel×Photoperiod interaction (*F*_2_,_716_=8.61, *P*<0.001) emerged as the second strongest predictor of biomass, indicative of metabolic effects of optimal light-night duration within each diel (Supplementary Fig. S5B; Supplementary Table S2).

The importance of noctoperiod duration over photoperiod length or light intensity in driving productivity gains remains largely unexplored in crop production, but this work establishes a foundation for mechanistic investigation into how extended dark periods can unlock yield potential.

Our findings probably reflect the fundamental role of the circadian clock in coordinating nocturnal carbon metabolism (Lu *et al.*, 2005; Graf *et al.*, 2010; Pilkington *et al.*, 2015). The circadian clock regulates both the rate of nocturnal starch degradation (Graf and Smith, 2011; Izumi, 2019) and the timing of growth-promoting processes to ensure reserves are neither depleted prematurely nor left unused by dawn. In Arabidopsis, circadian-gated growth-promoting factors such as PIF3 and PIF5 accumulate during the night and reach maximal activity at dawn, coinciding with the completion of starch degradation (Nozue *et al.*, 2007, 2011; Soy *et al.*, 2012). When comparing yield within the same photoperiod, extended dark hours, under 27 h cycles, may better synchronize these processes with the slower internal rhythms of long-clock lettuce, optimizing resource allocation for growth. Thus, the circadian resonance benefits reported in our study might occur due to proper alignment of dark-regulated metabolic processes with internal timing.

### Restoration of developmental timing and fitness implications

Our results reveal that the delayed bolting characteristic of long-clock lettuce under field and greenhouse conditions (24 h days) is reversed under resonant conditions (Fig. 7B). When long-clock plants were grown under 27 h cycles, their bolting frequency became comparable with that of normal-clock varieties, which are considered early bolting genotypes under standard long-day cultivation. This restoration of reproductive timing suggests that delayed bolting in long-clock lettuce results from circadian misalignment rather than inherent developmental reprogramming.

Under standard 24 h cultivation, the mismatch between the 27 h internal rhythms and 24 h environmental cycles of the plants might disrupt reproductive signalling cascades, inadvertently extending the vegetative phase. In our work, we show that when this temporal dissonance is eliminated through 27 h cycles, natural developmental progression resumes, and plants transition to reproduction at rates comparable with early bolting varieties.

The underlying mechanism probably involves realignment of photoperiodic flowering pathways, such as the CO–FT signalling module. CONSTANS (CO) protein accumulation must coincide with light periods to activate *FLOWERING LOCUS T* (*FT*) expression to initiate the reproductive programme (Sawa *et al.*, 2008). Under 24 h cycles, the 27 h internal rhythm may prevent proper coincidence, delaying bolting initiation.

This demonstrates that circadian resonance enhances fitness through multiple pathways: both by increased biomass accumulation and by faster reproductive development; consistent with observations in other organisms (Ouyang *et al.*, 1998; Beaver *et al.*, 2002).

### Normal-clock varieties show no circadian resonance effect

Our normal-clock subset of accessions includes winter cultivars developed for low-light greenhouse production (Table 1). These cultivars showed an overall lower biomass accumulation than long-clock accessions (Supplementary Fig. S2). In these accessions, we detected an enhanced performance under longer dark periods (7 h versus 4 h), independent of cycle length, when photoperiod was extended to 20 h (20:4 lowI and 20:7 lowI treatments) (Supplementary Fig. S6B). While 27 h cycles represent circadian dissonance for 24 h varieties (expected to reduce performance), our 27 h treatments necessarily provided longer nights (7 h versus 4 h) to maintain equivalent DLI. For winter cultivars adapted to extended nocturnal periods, this longer dark phase may be intrinsically beneficial, masking or counteracting circadian dissonance effects. This interpretation is supported by normal-clock accessions showing the strongest response specifically when dark periods were longest and light intensity was reduced (20:7 lowI treatment). Specifically, these accessions accumulated more biomass under 20:7 lowI than under 20:4 lowI and under 20:7 than under 20:4. However, neither 20:7 nor 20:7 lowI yielded higher biomass than 16:8 (Supplementary Fig. S6B).

Together, these results highlight again the key role of night duration for biomass accumulation, which in the case of normal-clock accessions might mask circadian resonance effects. Thus, future research should separate dark period duration from circadian alignment by testing additional treatments that isolate individual effects.

### Implications for controlled-environment agriculture

The ability to manipulate light cycles gives CEA a unique opportunity to apply circadian resonance strategies. Our findings show immediate practical benefits: we can create cultivar-specific lighting protocols that boost yield by matching biological rhythms rather than by adding more light. Vertical farms can switch to 27 h cycles for long-clock cultivars without any infrastructure changes, just by reprogramming the lights to obtain 11–29% more yield. This is particularly valuable in an industry focused on reducing costs.

Importantly, the strongest results came from Frillice, a commercial cultivar that is currently grown in vertical farms. Under 27 h cycles, Frillice produced up to 47% more biomass compared with standard 24 h cycles and also bolted earlier (Fig. 8A; Supplementary Fig. S15C). This combination—more growth under extended cycles plus faster development—tells us that Frillice probably has a long internal clock, although this remains to be tested. In any case, the yield increase observed in Frillice is highly significant and highlights an immense and underexplored yield potential not examined until now.

Ultimately, these results show that that our approach works on commercial varieties already in production, meaning that growers could apply circadian resonance to crops they are already growing and obtain highly significant yield increases.

## Conclusion

In conclusion, this work establishes circadian resonance as a transformative strategy for sustainable lettuce yield enhancement in CEA. By demonstrating that significant productivity gains (11–29%) can be achieved through biological rhythm optimization rather than increased resource inputs, we explore a new horizon for crop improvement that had remained unexplored thus far. Requiring only lighting schedule reprogramming in existing facilities, this strategy is ready for rapid applicability in CEA settings. More broadly, our findings challenge the conventional paradigm that yield improvements require proportional increases in energy, or extensive breeding programmes. In fact, our findings open up new research directions at the intersection of chronobiology, CEA, and sustainable food production that have direct applicability.

## Supplementary Material

erag222_Supplementary_Data

## Data Availability

All data collected, presented, and analyzed in this study are available as Supplementary tables accompanying this article.

## Supplementary data

The following supplementary data are available at *JXB* online.

Fig. S1. Cultivation setup and plant layout in the vertical farming experiment.

Fig. S2. Biomass accumulation comparison across all treatments grouped by circadian clock type.

Fig. S3. Representative long-clock lettuce morphologies under resonant (T27) and non-resonant conditions (T24) and different photoperiods after 30 cycles with the same daily light integral.

Fig. S4. Effect of diel cycle duration on time-corrected and area-corrected yield in long- and normal-clock lettuce accessions under equal daily light integrals.

Fig. S5. Statistical model development and validation for circadian resonance analysis.

Fig. S6. Fresh and dry weight of lettuce accessions with long and normal circadian clocks under six photoperiod treatments after 30 diel cycles.

Fig. S7. Fresh weight accumulation of lettuce accessions with long and normal circadian clocks under different photoperiod and day-length treatments.

Fig. S8. Fresh weight accumulation of all lettuce accessions grown under 24 h and 27 h days under three photoperiod combinations.

Fig. S9. Fresh weight biomass broken down per accessions with long, normal, and unknown circadian clocks under all the tested treatments.

Fig. S10. Fresh weight accumulation of cultivated lettuce accessions with long and normal circadian clocks under 20 h photoperiods at two day-length treatments.

Fig. S11. Top-down pictures of representative plants grown under 16:8 and 20:4 light:dark cycles.

Fig. S12. Morphology comparison between standard 16:8 treatment and high-intensive energy treatment 20:7.

Fig. S13. Tipburn severity scores across accessions and treatments after 30 diels.

Fig. S14. Percentage of bolted lettuce plants under different photoperiod and light treatments.

Fig. S15. Bolting incidence in cultivated lettuce by circadian clock type and treatment across harvest timepoints.

Fig. S16. Fresh weight accumulation of Frillice and Rabello lettuce cultivars under varying diel cycles after 30 d.

Fig. S17. Rabello lettuce exhibits morphological plasticity in response to photoperiod treatments, despite stable yield.

Table S1. Raw dataset with all phenotypic observations collected.

Table S2. Mixed-effects model statistics for fresh weight analysis under the same DLI treatments.

Table S3. Fresh weight means per accession under all treatments tested.

Table S4. Fresh weight means statistical comparisons for each accession under all treatments.
