# Supplementary material for: Matching circadian rhythms to light–dark cycles increases lettuce yield by 29% in vertical farms without additional energy input
Source: J Exp Bot. 2026 May 12;77(16):5336–49. doi: 10.1093/jxb/erag222 (PMC13529320; doi:10.1093/jxb/erag222)
Supplement: Supplementary file 1 [file erag222_supplementary_data.zip › JEXBOT317959-file002.pdf]

Supplementary Information

Supplementary Figures S1-S17

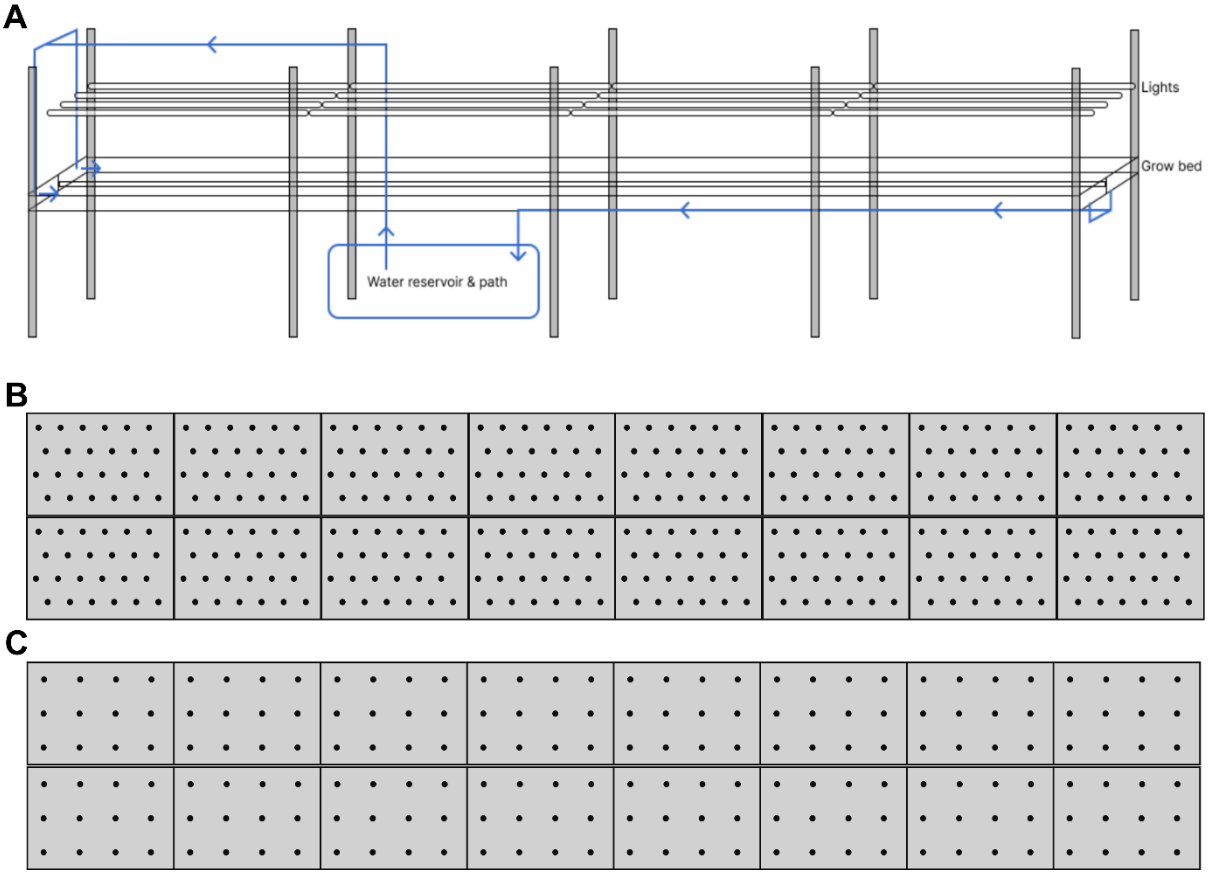

Fig. S1. Cultivation setup and plant layout in the vertical farming experiment. A. Scheme of a single rack level (from a total of five), illustrating the placement of lights, growing bed, and the water reservoir with the direction of water flow. Each horizontal layer was assigned a distinct experimental treatment. B. Planting layout in the floating rafts from diel 6 to diel 32, shown in top-down view. Black circles represent individual planting sites. C. Shows spaced positions after 32 diels and until 46.

ALT TEXT: Three-panel schematic of vertical farming setup. A is a perspective diagram of a single rack level showing horizontal LED light strips above a grow bed, with blue arrows indicating recirculating water flow through a reservoir below. B shows a top-down grid layout of two floating raft rows with densely spaced planting sites (48 plants per square metre) used from diel 6 to 32. C shows the same grid with sparser planting sites (24 plants per square metre) used from diel 32 to 46.

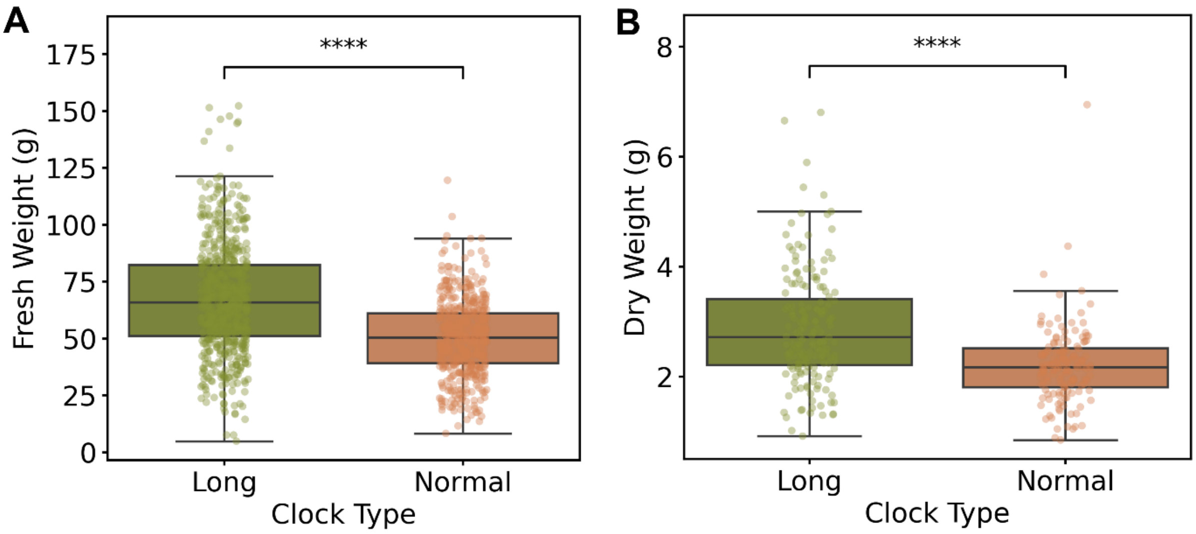

*Fig. S2. Biomass accumulation comparison across all treatments grouped by circadian clock type. A. Fresh weight (FW) after 30 diels in long (n = 611) and normal-clock (n = 492) plants B. dry weight (DW) after 30 diels of lettuce accessions with long (~27 h; olive. n = 171) and normal (~24 h; orange, n = 140) circadian clocks, pooled across all photoperiod and day-length treatments. Each dot represents an individual plant. Asterisks indicate statistically significant differences based on a Mann–Whitney U test (**** p < 0.0001).*

ALT TEXT: Two box plots with overlaid individual data points comparing long-clock (olive) and normal-clock (orange) lettuce accessions pooled across all treatments. A shows fresh weight in grams; long-clock median approximately 68 grams versus normal-clock approximately 50 grams. B shows dry weight in grams; long-clock median approximately 2.8 grams versus normal-clock approximately 2.2 grams. Both comparisons are statistically significant (p less than 0.0001).

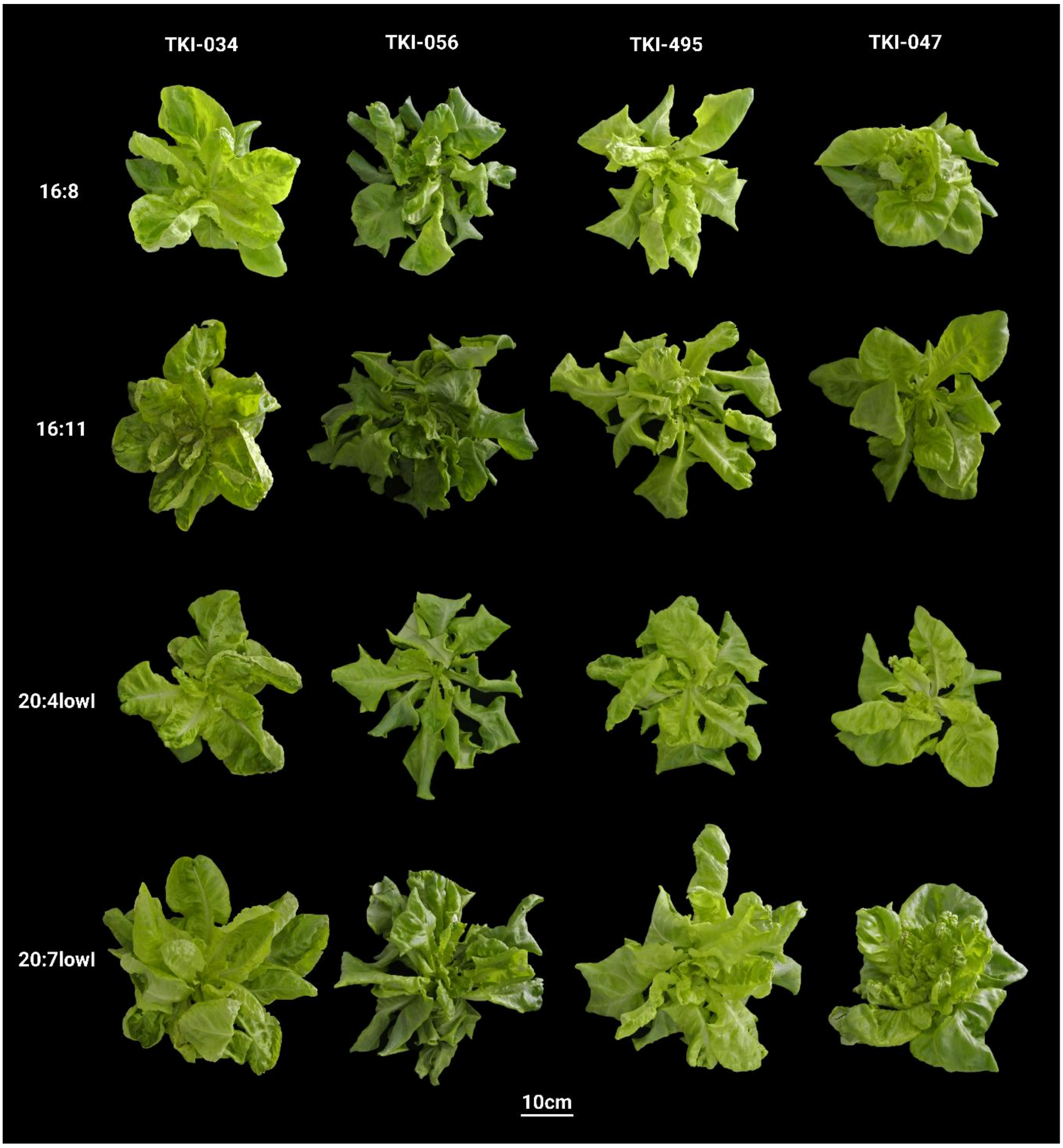

Fig. S3. Representative long-clock lettuce morphologies under resonant (T27) and non-resonant conditions (T24) and different photoperiods after 30 cycles with the same daily light integral. Top-down images of representative long-clock lettuce accessions (TKI-034, TKI-056, TKI-495, TKI-047) grown under four different photoperiod regimes with the same daily light integral (DLI = 14.4 mol m $^{-2}$ d $^{-1}$): 16:8, 16:11, 20:4 lowl, and 20:7 lowl. Plants grown under circadian-resonant conditions (16:11 and 20:7 lowl) appear larger and more vigorous compared to their 24-hour homologous treatments (16:8 and 20:4 lowl), supporting the positive effect of circadian alignment on biomass accumulation. A 10 cm scale bar is provided for size reference.

ALT TEXT: Grid of top-down photographs of four long-clock lettuce accessions (TKI-034, TKI-056, TKI-495, TKI-047) grown under four equal daily light integral treatments: 16:8, 16:11, 20:4 lowl, and 20:7 lowl. Plants under 27-hour resonant treatments (16:11 and 20:7 lowl) visibly show larger rosettes than their 24-hour counterparts. Scale bar 10 centimetres.

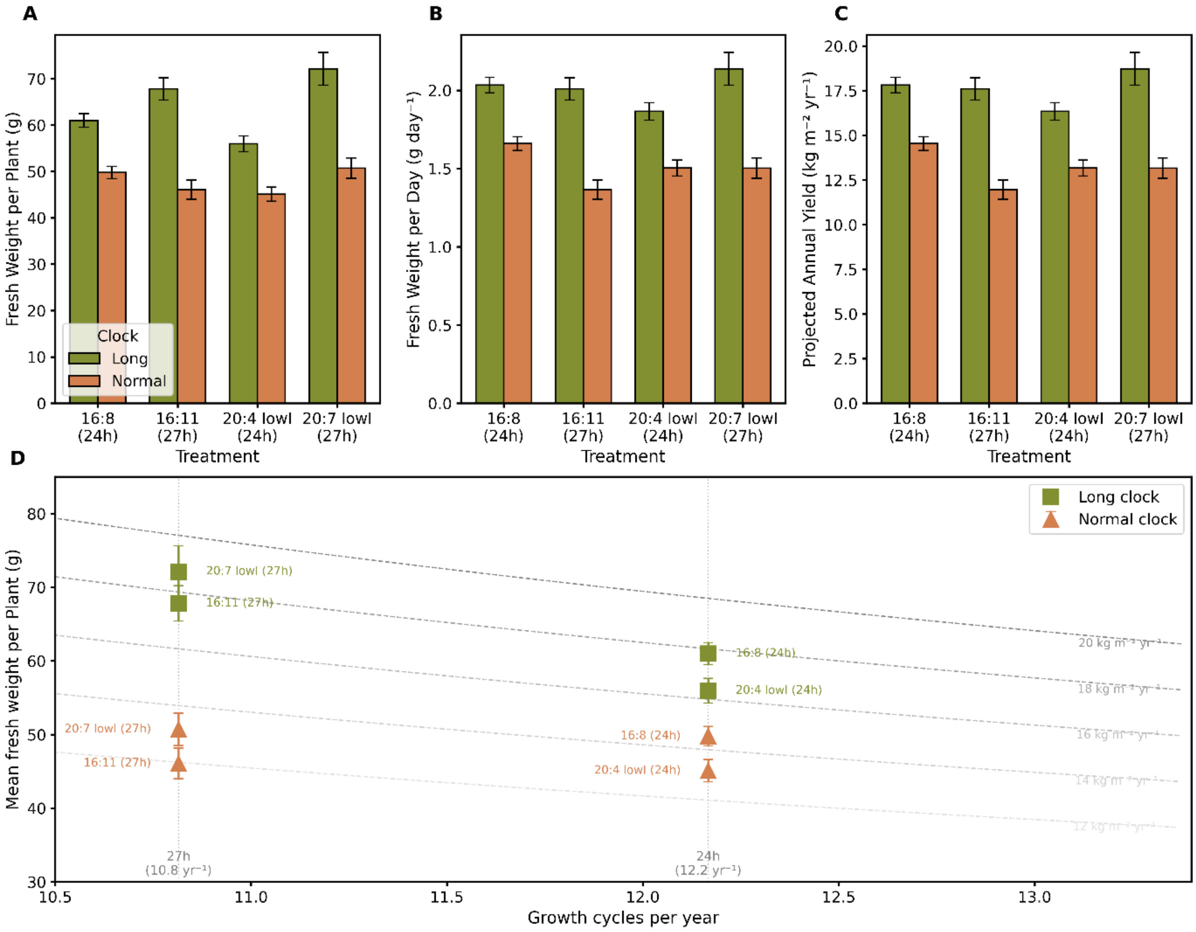

Fig. S4. Effect of diel cycle duration on time-corrected and area-corrected yield in Long and Normal clock lettuce accessions under equal daily light integrals. A potential concern with extending diel cycle length from 24h to 27h is the reduction in annual rotation frequency: 27h cycles yield 10.8 crop rotations per year compared to 12.2 under 24h cycles, an 11% decrease. To assess whether the biomass gains observed in Long clock accessions under 27h cycles are sufficient to offset this reduced throughput, we calculated time-corrected (fresh weight per day) and area-corrected (projected annual yield per m^2) metrics for all equal-DLI treatments. A. Mean fresh weight per plant after 30 diel cycles under four equal-DLI treatments. B. Fresh weight normalised by the actual number of elapsed days (30 days for 24h cycles; 33.75 days for 27h cycles), reflecting daily biomass accumulation rate. C. Projected annual yield per unit growing area, calculated as mean daily fresh weight × 365 × 24 plants m^{-2}, accounting for the difference in rotation frequency between diel cycle lengths (12.2 vs 10.8 rotations yr^{-1} for 24h and 27h cycles, respectively). D. Iso-yield plot relating mean fresh weight per plant to growth cycles per year. Dashed grey curves represent lines of equal projected annual yield (kg m^{-2} yr^{-1}); points above a given curve yield more annually than its value. Dotted vertical lines indicate the number of cycles completed per year under 24h (12.2 yr^{-1}) and 27h (10.8 yr^{-1}) diel cycles. Long clock accessions under 27h cycles (left cluster) fall on equivalent or higher iso-yield curves than the same accessions under 24h cycles (right cluster), demonstrating that the 11% reduction in annual rotation frequency is fully offset by increased biomass per plant. Normal clock accessions show no such compensation. Error bars represent ±1 standard error of the mean. Olive squares: Long clock (~27h period); orange triangles: Normal clock (~24h period).

ALT TEXT: Four-panel figure showing time-corrected and area-corrected yield analysis. A shows mean fresh weight per plant across four equal daily light integral treatments, with long-clock (olive) consistently higher than normal-clock (orange). B shows fresh weight per day, with long-clock values maintained or higher under 27-hour treatments. C shows projected annual yield per square metre, with long-clock accessions under 27-hour cycles matching or exceeding their 24-hour yield. D is an iso-yield

scatter plot with growth cycles per year on the x-axis and mean fresh weight on the y-axis; dashed grey curves represent equal annual yield in kilograms per square metre per year; long-clock squares cluster at 10.8 cycles on higher iso-yield curves than their 24-hour counterparts at 12.2 cycles, while normal-clock triangles show no such trend.

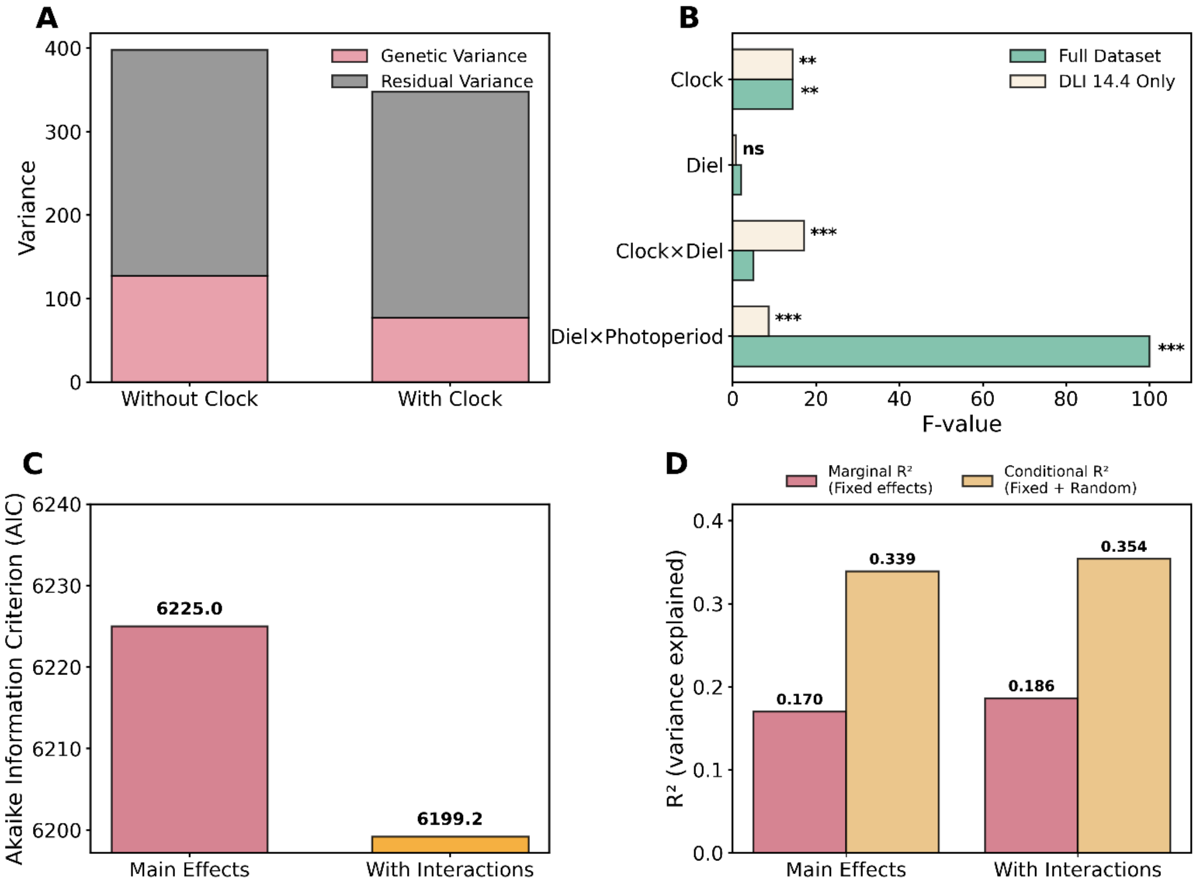

*Fig. S5. Statistical model development and validation for circadian resonance analysis. A. Variance components in preliminary models with and without clock information as a fixed effect. Including clock as a fixed effect reduced unexplained genetic variance (pink) by 47%, demonstrating that the circadian clock explains substantial variation previously attributed to general accession differences. B. Factor importance (F-values) comparing full dataset versus DLI 14.4 subset, illustrating the rationale for comparing treatments with same DLI. In the full dataset, DLI dominates variance explanation (F = ~100), overshadowing other biological effects. When comparing treatments with equivalent DLI (14.4 subset), Clock, Photoperiod, and Noctoperiod effects become clearly detectable and biologically interpretable. C. Model selection via Akaike Information Criterion (AIC) comparison. Including Clock × Diel interaction significantly improved model fit over main effects alone (without the interaction) ΔAIC = 25.8, χ^2 = 29.81, df = 2, p < 0.001) justifying the inclusion of circadian resonance terms in the final statistical model. D. Model performance (R^2) improvement through iterative model development. The final model with Clock × Diel interaction explained 35.4% of total variance (conditional R^2), with fixed effects alone explaining 18.6% (marginal R^2). Our last mixed-effects models used maximum likelihood estimation (REML = FALSE) with Accession as random intercept to account for genetic background variation (n = 732 observations, 16 cultivated lettuce accessions with known circadian clock). Type III ANOVA with Satterthwaite's method assessed fixed effect significance. Model comparison used likelihood ratio tests. Significance levels: * p < 0.05, ** p < 0.01, *** p < 0.001, ns = not significant.* See Table S2 for further details.

ALT TEXT: Four-panel figure showing statistical model development. A shows stacked bar charts of genetic and residual variance with and without clock as a fixed effect; including clock reduces genetic variance by 47 percent. B shows horizontal bar charts of F-values for four model terms comparing the full dataset (teal) and the daily light integral 14.4 subset (beige); daily light integral dominates in the full dataset but clock and interaction terms become detectable in the subset. C shows two bars comparing Akaike Information Criterion values of 6225.0 for main effects only and 6199.2 with interactions, supporting inclusion of interaction terms. D shows paired bars of marginal and conditional R-squared

for main effects and full interaction models; the final model explains 18.6 percent marginal and 35.4 percent conditional variance.

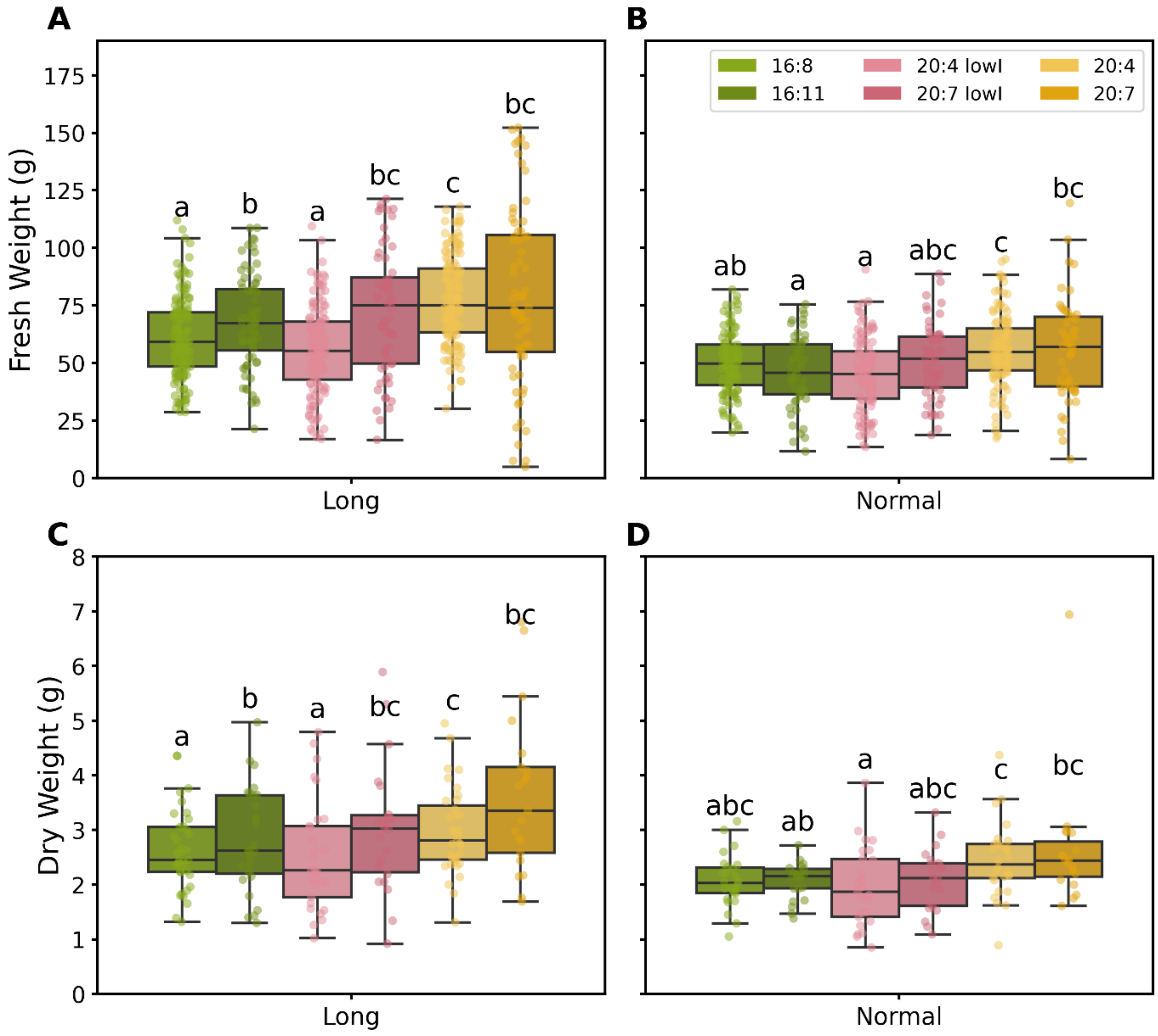

Fig. S6. Fresh and dry weight of lettuce accessions with long and normal circadian clocks under six photoperiod treatments after 30 diel cycles. A–B Fresh weight (FW) of accessions with long (~27 h) and normal (~24 h) circadian clocks grown under 16 h light/8 h dark (16:8, light green), 16 h light/11 h dark (16:11, dark green), 20 h light/4 h dark at standard intensity (20:4, beige), 20 h light/7 h dark at standard intensity (20:7, gold), and reduced-intensity variants of the 20-hour treatments matched to the 16-hour daily light integral (20:4 lowI, light rose; 20:7 lowI, dark rose). C–D. Dry weight (DW) of the same accessions and treatments. T27 harvests were performed at 33.75 days, equivalent to 30 diel cycles under a 27-hour period. Statistical comparisons were performed separately within each clock group using a rank-transformed linear model, followed by estimated marginal means and FDR-adjusted pairwise comparisons. Letters indicate statistically significant differences among treatments ($p < 0.05$); treatments sharing a letter are not significantly different.

ALT TEXT: Four-panel figure (A to D) showing fresh weight (A, B) and dry weight (C, D) box plots with individual data points for long-clock (A, C) and normal-clock (B, D) accessions across all six treatments at 30 diel cycles. Long-clock accessions show significantly higher biomass under high-energy 20:4 and 20:7 treatments; normal-clock accessions show fewer significant differences among treatments.

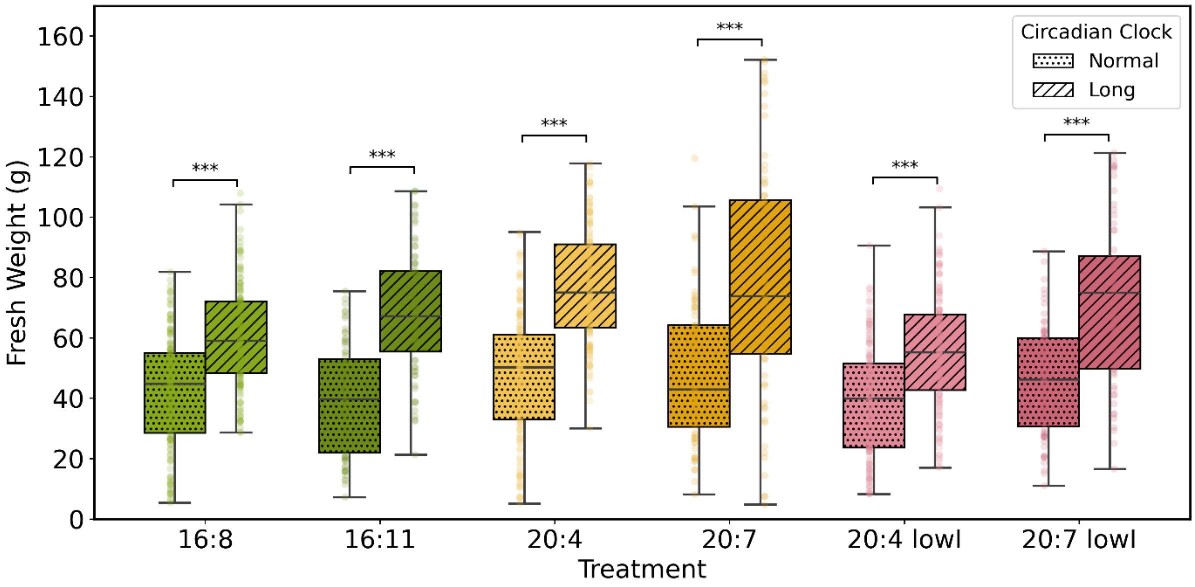

*Fig. S7. Fresh weight (FW) accumulation of lettuce accessions with long (~27 hours) and normal (~24 hours) circadian clocks under different photoperiod and day-length treatments. Fresh weight was measured after 30 diel cycles (24 hours) or the equivalent in 27 hours' time (34 days). Statistical comparisons are performed comparing all accessions having a normal clock (dotted box) vs all accessions having a long clock (dashed box). Asterisks indicate statistically significant differences based on a Mann–Whitney U test (*** p < 0.0001).*

ALT TEXT: Grouped box plot showing fresh weight in grams across six treatments, comparing normal-clock (dotted fill) and long-clock (hatched fill) accessions pooled together. Long-clock accessions show significantly higher fresh weight than normal-clock accessions in all six treatments (p less than 0.0001 in each case).

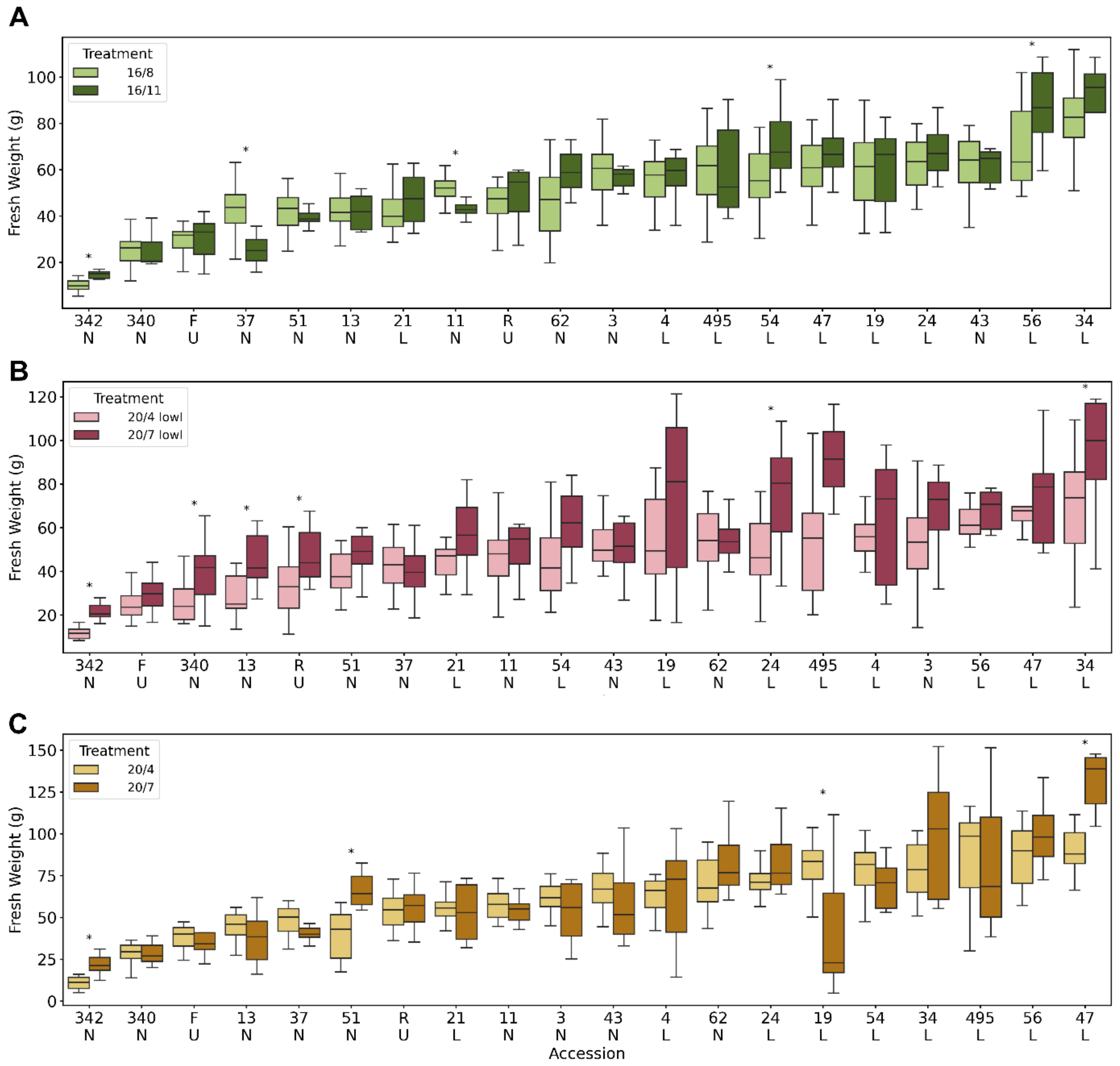

Fig. S8. Fresh weight (FW) accumulation of all lettuce accessions grown under 24-hour and 27-hour days under 3 photoperiod combinations. A. Plants grown under standard cultivation conditions of 16 h light/8 h dark (16:8, 250 µmol /s light) but also under 27-hour days of 16 h light/11 h dark. The x axis indicates accession number and N/L/U whether that accession has a normal, long or unknown circadian clock. B-C. Longer photoperiod screenings (20 hours) in which we supplied 4 hours of dark or 7 hours (20:4, 20:7). B. shows this longer photoperiod treatment at a reduced intensity (200 µmol/s light) to provide the same DLI as the 16-hour treatments, in C intensity is kept standard (250 µmol/s) and consequently higher light energy is used. Asterisks denote significant differences in pairwise comparison for each accession under different treatments. (p < 0.05).*

ALT TEXT: Three-panel figure (A to C) showing fresh weight per accession ordered by biomass. A compares 16:8 (light green) versus 16:11 (dark green) across 20 accessions labelled with clock type (N, L, or U). B compares 20:4 lowl (light rose) versus 20:7 lowl (dark rose). C compares 20:4 (light gold) versus 20:7 (dark gold). Asterisks indicate significant pairwise differences within accessions. Long-clock accessions (L) generally show higher biomass.

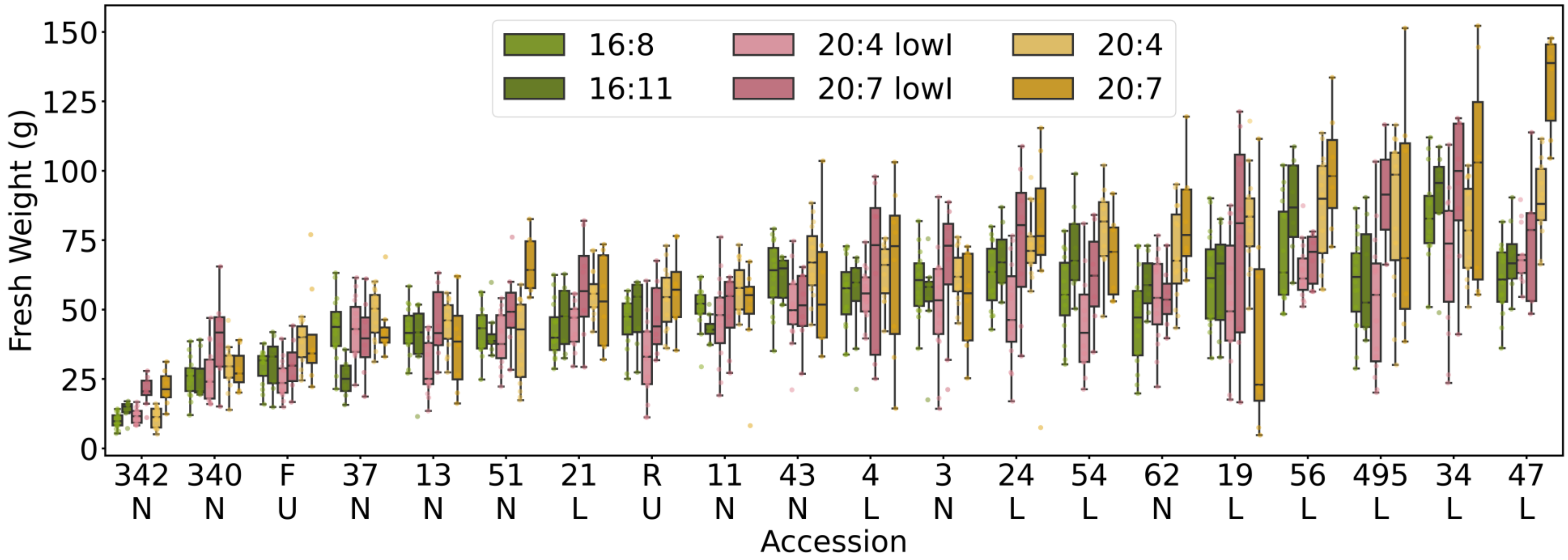

Fig. S9. Fresh weight biomass broken down per accessions with long (~27 h, L), normal (~24 h, N) and unknown (U) circadian clocks under all the tested treatments. Plants were grown under six environmental conditions: 16 h light/8 h dark (16:8, green) and 20 h light/4 h dark (20:4, beige) on 24-hour days; 16 h light/11 h dark (16:11, dark green) and 20 h light/7 h dark (20:7, gold) on 27-hour days; and reduced light intensity variants of 20:4 and 20:7 ("lowI", light rose and dark rose respectively) to match daily light integral (DLI) with 16 hours treatments. Statistical comparisons were performed separately within each clock group using a linear model, followed by estimated marginal means and FDR-adjusted. Pairwise comparisons are available in Table S4.

ALT TEXT: Single wide box plot showing fresh weight in grams for all 20 accessions across six treatments, ordered by increasing biomass from left to right. Accessions are labelled by number and clock type (N, L, U) on the x-axis. Long-clock accessions (L) cluster toward the right with consistently higher fresh weight; high-energy 20:4 and 20:7 treatments (gold) show the highest values across most accessions.

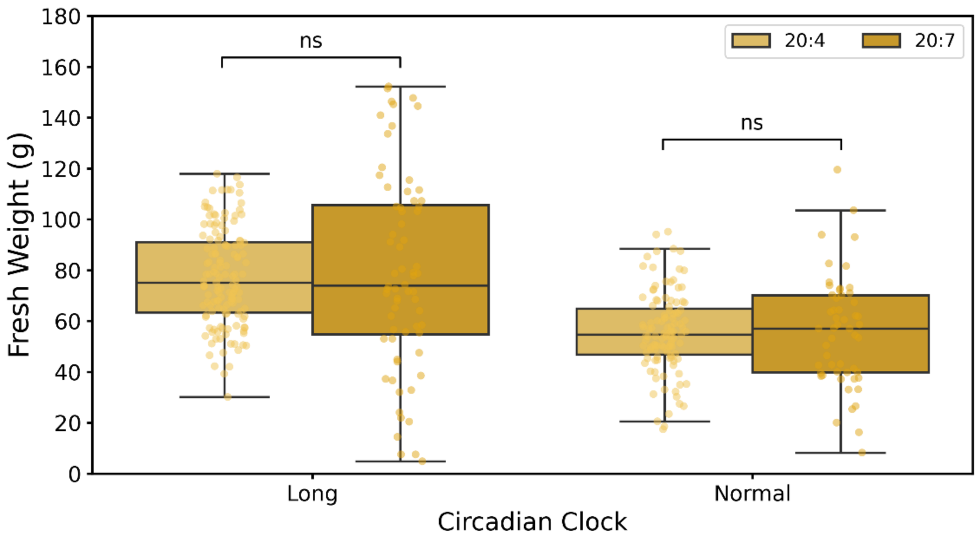

Fig. S10. Fresh weight (FW) accumulation of cultivated lettuce accessions with long (~27 hours) and normal (~24 hours) circadian clocks under 20-hour photoperiods at two day-length treatments. Plants were grown under 20 h light/4 h dark (20:4, beige) on 24-hour days and 20 h light/7 h dark (20:7, gold) on 27-hour days with a standard light intensity (250 μmol/m^2·s). Fresh weight was measured after 30 diel cycles. ns indicates statistically non-significant differences among treatments.

ALT TEXT: Box plot comparing fresh weight under 20:4 (light gold) and 20:7 (dark gold) standard-intensity treatments for long-clock and normal-clock accessions. No significant differences are detected between treatments in either clock type (both comparisons labelled ns).

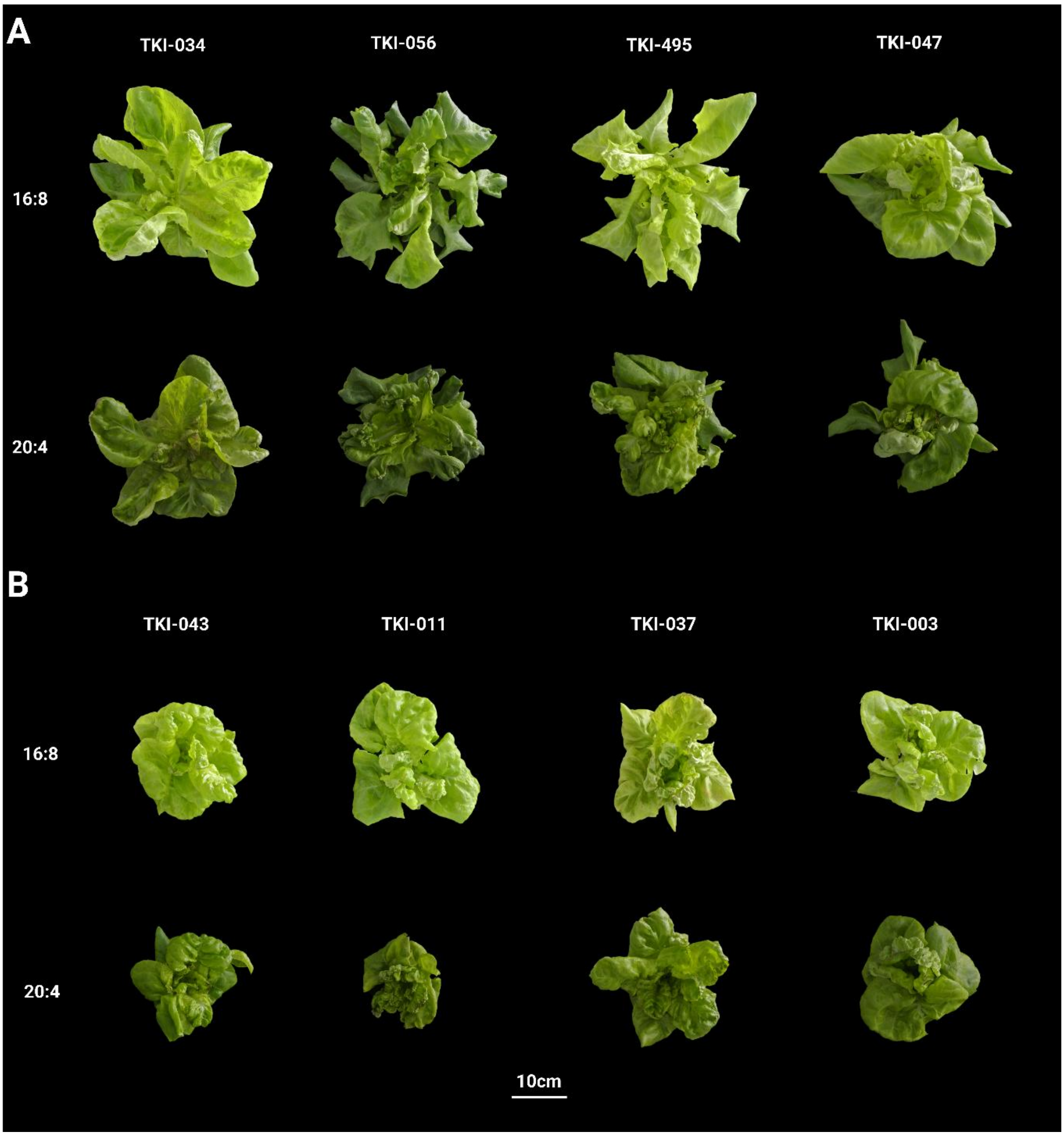

Fig. S11. Top-down pictures of representative plants grown under 16:8 and 20:4 light:dark cycles. A. Accessions with a long circadian period (TKI-034, TKI-056, TKI-495, TKI-047) and B. Accessions with a normal (~24 h) circadian period (TKI-043, TKI-011, TKI-037, TKI-003). Plants exposed to the 20:4 photoperiod show visible morphological alterations compared to those under 16:8 (as shown in Fig. S3), including darker leaf coloration, increased curliness, and irregular rosette structure. A scale bar of 10 cm is included for size reference.

ALT TEXT: Two-panel figure (A and B) showing top-down photographs of lettuce plants on a black background under 16:8 and 20:4 treatments. A shows four long-clock accessions (TKI-034, TKI-056, TKI-495, TKI-047); plants under 16:8 have open, spreading rosettes while under 20:4 leaves appear darker, curlier, and the rosette structure is more irregular. B shows four normal-clock accessions (TKI-043, TKI-011, TKI-037, TKI-003) with similar morphological changes under 20:4. Scale bar 10 centimetres.

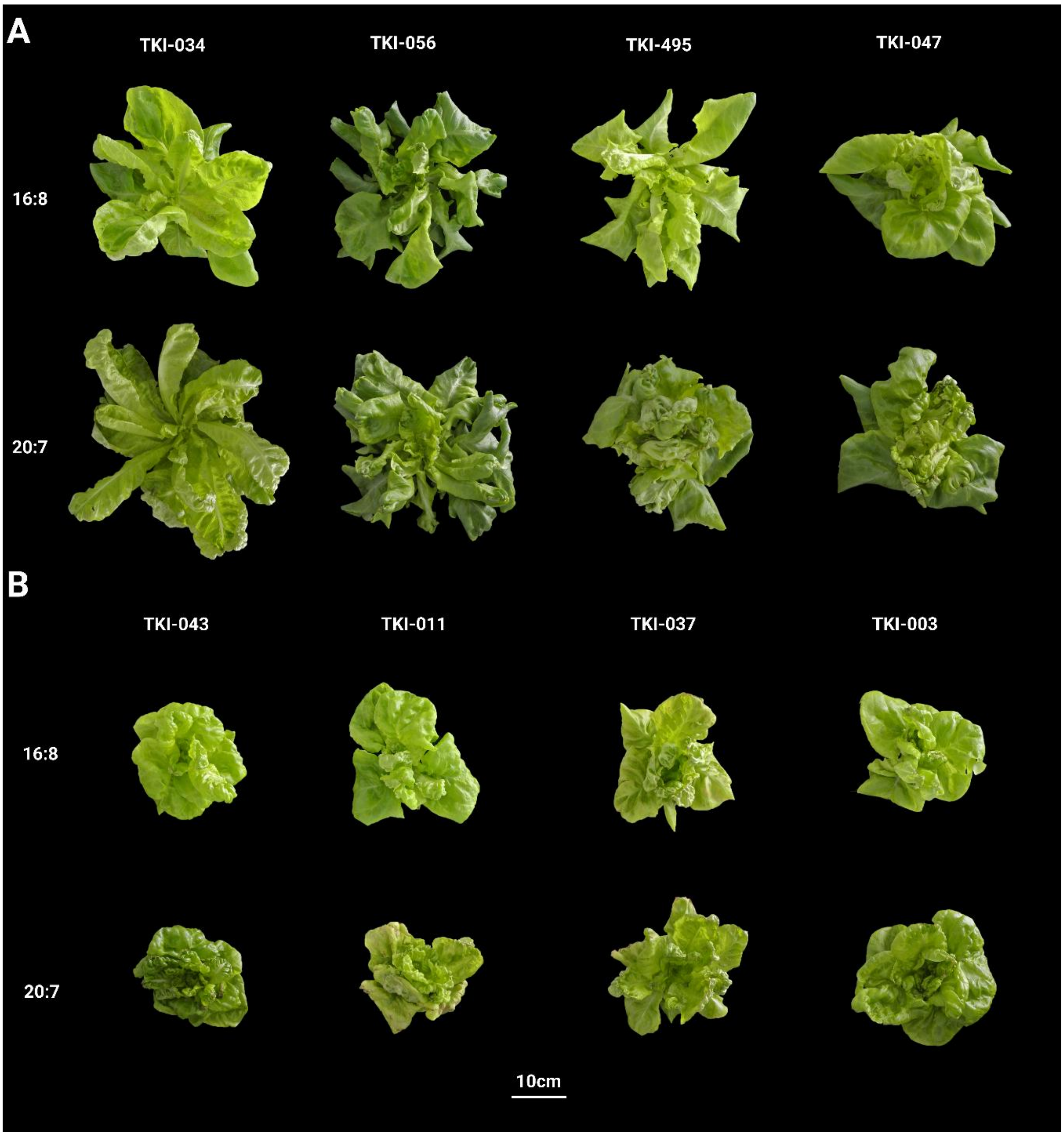

*Fig. S12. Morphology comparison between standard 16:8 treatment and high-intensive energy treatment 20:7. Top-down pictures of representative plants grown under 16:8 and 20:7 light: dark cycles. A. Accessions with a long circadian period (TKI-034, TKI-056, TKI-495, TKI-047) under 16:8 (*as shown in Fig. S3A and Fig. 11A) *and under 20:7. B. Accessions with a normal (~24 h) circadian period (TKI-043, TKI-011, TKI-037, TKI-003) under 16:8 (as shown in Fig. S3B and Fig. S11B) compared to 20:7. Plants exposed to the 20:7 photoperiod show increased curliness, and irregular rosette structure. A scale bar of 10 cm is included for size reference.*

ALT TEXT: Two-panel figure (A and B) showing top-down photographs of lettuce plants on a black background under 16:8 and 20:7 standard-intensity treatments. A shows four long-clock accessions (TKI-034, TKI-056, TKI-495, TKI-047); plants under 20:7 appear larger but with more spreading, irregular rosettes compared to 16:8. B shows four normal-clock accessions (TKI-043, TKI-011, TKI-037, TKI-003); under 20:7 plants appear more compact with curled, irregular leaves. Scale bar 10 centimetres.

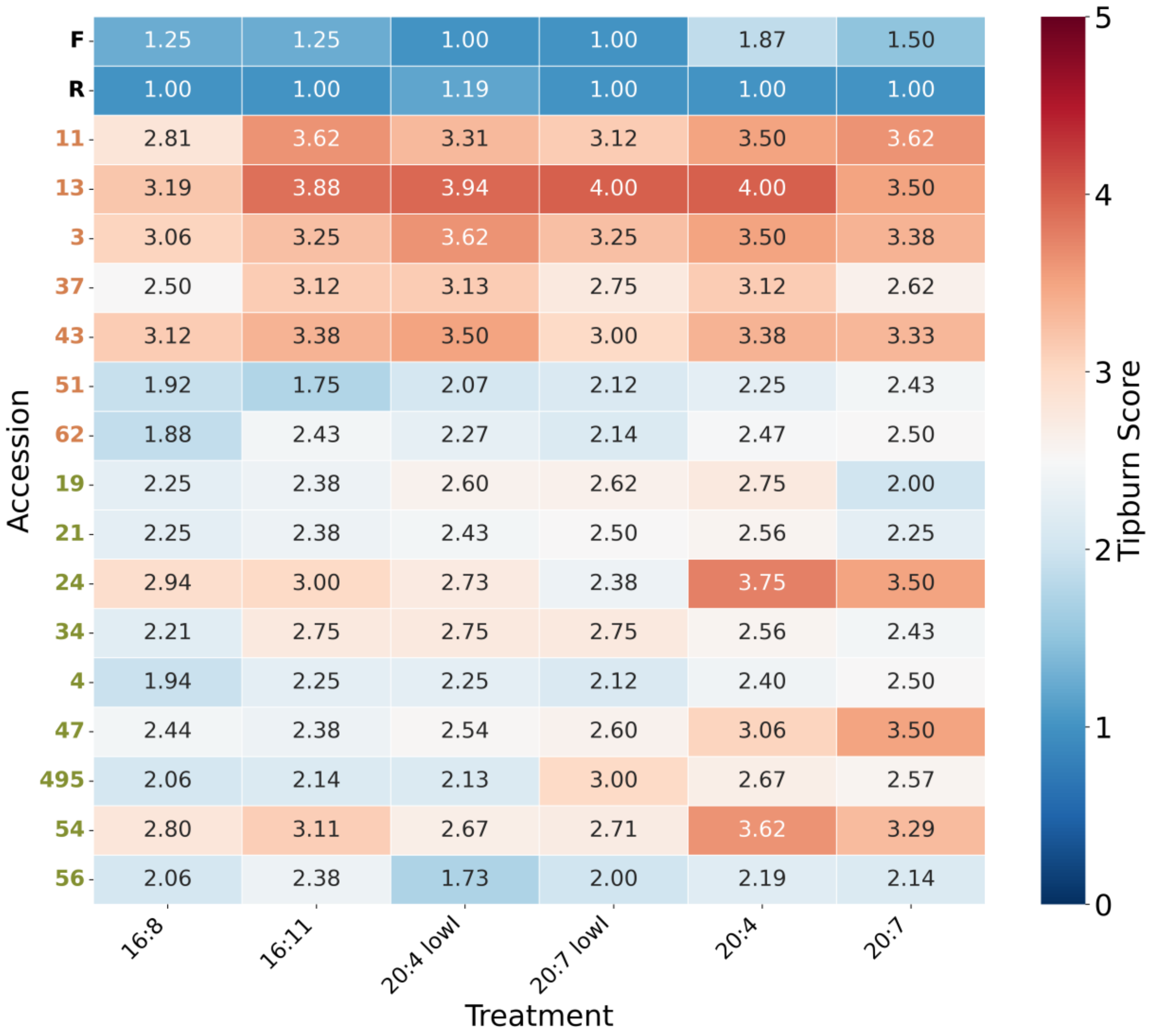

Fig. S13. Tipburn severity scores across accessions and treatments after 30 diels. Heatmap showing average tipburn severity (scale 0–5) for each lettuce accession (N≥8) under six photoperiod and day-length treatments. Accessions with long (~27 h) circadian clocks are labelled in olive, and those with normal (~24 h) clocks in orange, on the y axis. Two commercial cultivars, R and F, are also included and labelled in black. A score of 0 indicates no tipburn, while 5 represents the most severe damage observed, indicated by the scale bar.

ALT TEXT: Heatmap showing mean tipburn score (0 to 5) for 18 accessions across six treatments. Colour scale ranges from blue (score 0) to dark red (score 5). Commercial cultivars F and R (black labels, top) consistently score below 2. Normal-clock accessions (orange labels) show predominantly high scores of 3 to 4. Long-clock accessions (olive labels) show more variable scores, generally between 2 and 3, with some exceptions under high-energy treatments.

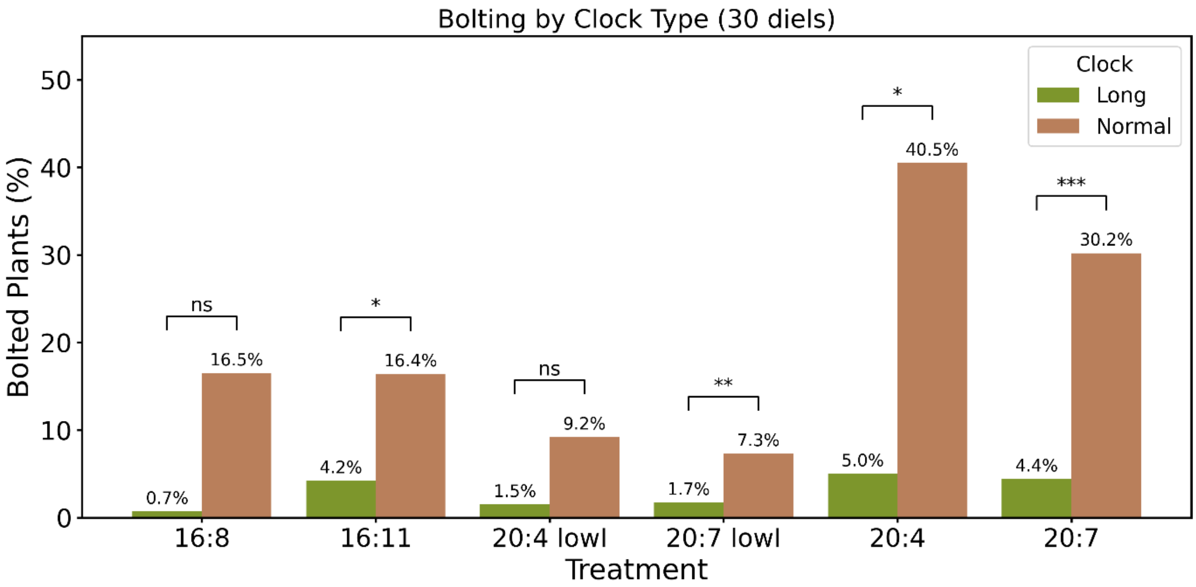

Fig. S14. Percentage of bolted lettuce plants under different photoperiod and light treatments. Bolting incidence was evaluated in accessions with long (~27 h; olive) and normal (~24 h; orange) circadian clocks after 30 diel cycles. Bars represent the percentage of plants that bolted under each treatment. Treatments included 24 hours (16:8, 20:4), 27 hours (16:11, 20:7) and reduced light intensity 24-hour and 27-hour cycles (20:4 lowl, 20:7 lowl). Significant differences between matched 24-hour vs 27-hour daylength treatments (e.g., 20:4 vs 20:7) were assessed using Fisher's exact test. Asterisks denote significance levels: p < 0.05 (*), p < 0.01 (**), p < 0.001(***); ns indicates non-significant statistical difference.

ALT TEXT: Grouped bar chart showing bolting incidence at 30 diel cycles for long-clock (olive) and normal-clock (orange) accessions across six treatments. Long-clock bolting remains below 5 percent across all treatments. Normal-clock bolting ranges from 7.3 to 40.5 percent, with highest incidence under 20:4 (40.5 percent) and 20:7 (30.2 percent). Significant differences between clock types are detected under 16:11, 20:7 lowl, 20:4, and 20:7.

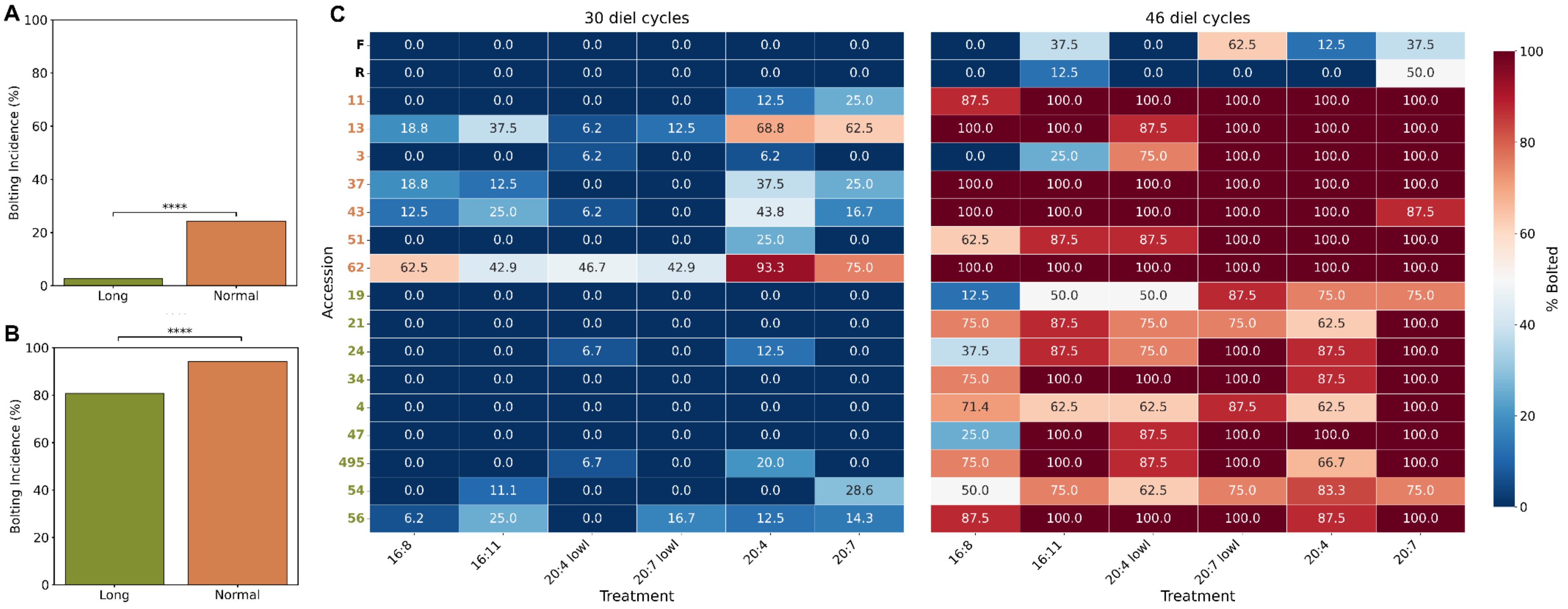

*Fig. S15. Bolting incidence in cultivated lettuce by circadian clock type and treatment across harvest timepoints. A-B. Overall bolting percentage in long-clock (green) and normal-clock (orange) lettuce accessions after 30 diel cycles (A) and 46 diel cycles (B), pooled across all treatments. Asterisks indicate statistically significant differences between clock types (Chi-squared test, **** $p < 0.0001$). C. Heatmap showing bolting percentage for individual accessions under six photoperiod treatments after 30 diel cycles (left panel) and 46 diel cycles (right panel). Treatments include: 16:8 (16h light/8h dark, 24-hour cycle), 16:11 (16h light/11h dark, 27-hour cycle), 20:4 lowl and 20:7 lowl (20-hour photoperiods with reduced light intensity to match daily light integral), 20:4 and 20:7 (20-hour photoperiods at standard light intensity). Accession numbers are color-coded by circadian clock type: green (long-clock, ~27 hours), orange (normal-clock, ~24 hours), and black (commercial cultivars with unknown clock periods). Colour scale represents bolting percentage from 0% (dark blue) to 100% (dark red). Long-clock accessions show minimal bolting at 30 cycles but increased bolting under 27-hour treatments by 46 cycles, while normal-clock accessions bolt extensively under most conditions by 46 cycles.*

ALT TEXT: Three-panel figure (A, B, C). A and B are bar charts showing overall bolting percentage pooled across all treatments at 30 diel cycles (A) and 46 diel cycles (B), comparing long-clock (olive) and normal-clock (orange); both comparisons are highly significant (p less than 0.0001). C shows two side-by-side heatmaps of bolting percentage per accession and treatment at 30 and 46 diel cycles; colour scale ranges from dark blue (0 percent) to dark red (100 percent). At 30 cycles most long-clock accessions show near-zero bolting; by 46 cycles nearly all accessions reach high bolting frequencies under most treatments.

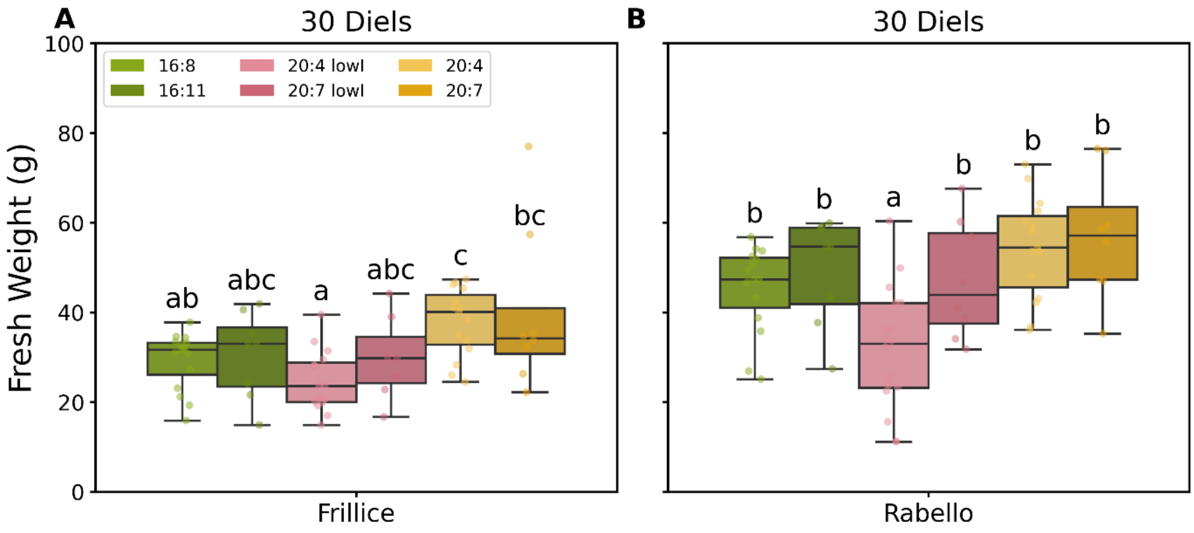

Fig. S16. Fresh weight accumulation of Frillice (A) and Rabello (B) lettuce cultivars under varying diel cycles after 30 days. Plants were grown under six environmental conditions on either 24-hour or 27-hour cycles: 16:8 (green) and 20:4 (beige) on 24-hour days; 16:11 (dark green) and 20:7 (gold) on 27-hour days; and reduced intensity variants of 20:4 and 20:7 (light rose and rose, respectively) to match the daily light integral of the 16-hour treatments. Fresh weight was recorded after 30 diel cycles. Statistical comparisons were conducted separately for each cultivar using a linear model for Rabello and a rank-transformed linear model for Frillice. Different letters indicate statistically significant differences among treatments ($p < 0.05$).

ALT TEXT: Two box plots (A and B) showing fresh weight in grams at 30 diel cycles for two commercial cultivars: Frillice (A) and Rabello (B) across six treatments. Frillice shows no significant treatment differences at this early timepoint, with all medians below 40 grams. Rabello shows significantly lower fresh weight under 20:4 lowI compared to other treatments, with medians around 50 grams for most treatments.

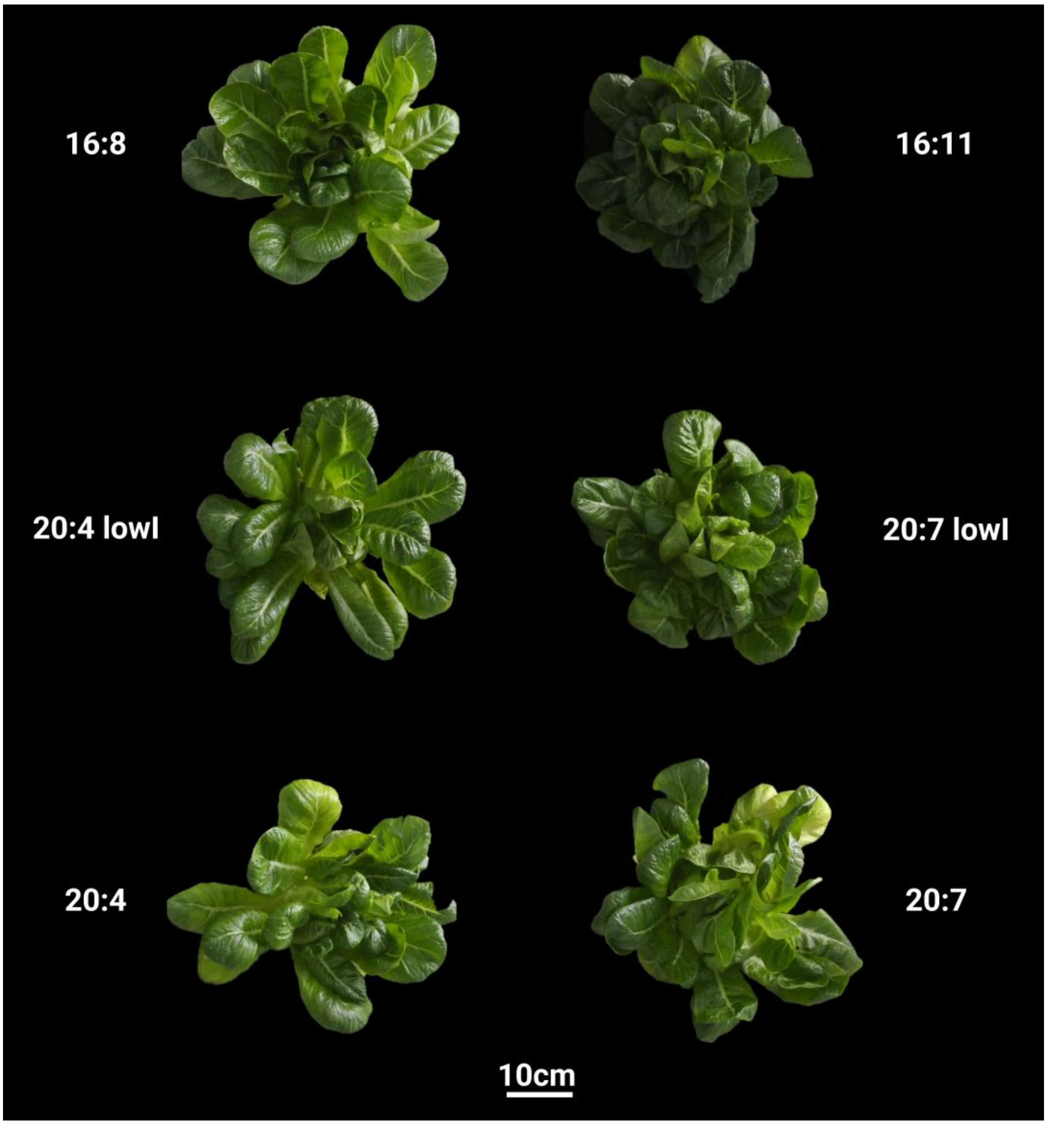

Fig. S17. Rabello lettuce exhibits morphological plasticity in response to photoperiod treatments, despite stable yield. Plants were grown for 46 diel cycles under either 24-hour (T24) or 27-hour (T27) days with varying photoperiods and light intensities. Treatments included 16:8, 20:4, and 20:4 low intensity (lowl) under T24, and 16:11, 20:7, and 20:7 lowl under T27 conditions. A 10 cm scale bar is provided for reference. While biomass accumulation remained consistent across treatments (Fig. 8), these top-view images highlight distinct changes in rosette morphology, suggesting high morphological responsiveness (plasticity) of Rabello to the supplied treatment.

ALT TEXT: Six top-down photographs of Rabello lettuce plants on a black background, one per treatment (16:8, 16:11, 20:4 lowl, 20:7 lowl, 20:4, 20:7), showing distinct rosette morphology across conditions despite similar biomass. Scale bar 10 centimetres.
